# Imagine the bright side of life: A randomized controlled trial of two types of interpretation bias modification procedure targeting adolescent anxiety and depression

**DOI:** 10.1371/journal.pone.0181147

**Published:** 2017-07-17

**Authors:** E. L. de Voogd, E. de Hullu, S. Burnett Heyes, S. E. Blackwell, R. W. Wiers, E. Salemink

**Affiliations:** 1 Department of Developmental Psychology, University of Amsterdam, Amsterdam, The Netherlands; 2 Department of Psychology and Educational Sciences, Open University, Zwolle, The Netherlands; 3 School of Psychology, University of Birmingham, Birmingham, United Kingdom; 4 MRC Cognition & Brain Sciences Unit, Cambridge, United Kingdom; 5 Mental Health Research and Treatment Center, Ruhr-Universität Bochum, Bochum, Germany; TNO, NETHERLANDS

## Abstract

**Introduction:**

Anxiety and depression are highly prevalent during adolescence and characterized by negative interpretation biases. Cognitive bias modification of interpretations (CBM-I) may reduce such biases and improve emotional functioning. However, as findings have been mixed and the traditional scenario training is experienced as relatively boring, a picture-based type of training might be more engaging and effective.

**Methods:**

The current study investigated short- and long-term effects (up to 6 months) and users’ experience of two types of CBM-I procedure in adolescents with heightened symptoms of anxiety or depression (N = 119, aged 12–18 year). Participants were randomized to eight online sessions of text-based scenario training, picture-word imagery training, or neutral control training.

**Results:**

No significant group differences were observed on primary or secondary emotional outcomes. A decrease in anxiety and depressive symptoms, and improvements in emotional resilience were observed, irrespective of condition. Scenario training marginally reduced negative interpretation bias on a closely matched assessment task, while no such effects were found on a different task, nor for the picture-word or control group. Subjective evaluations of all training paradigms were relatively negative and the imagery component appeared particularly difficult for adolescents with higher symptom levels.

**Conclusions:**

The current results question the preventive efficacy and feasibility of both CBM-I procedures as implemented here in adolescents.

## Introduction

Anxiety and depression are highly prevalent in the general population and often have their onset during adolescence [1]. Both anxiety and depression are associated with negative interpretation biases, which have been demonstrated in adults (for a review, see [2]), and also in adolescents specifically (e.g., [3], [4], [5]). Cognitive Bias Modification of Interpretations (CBM-I) is an experimental paradigm that has potential to be developed into a low-barrier early intervention for anxiety and depression. In CBM-I, interpretations are directly manipulated by relatively easy computer tasks involving ambiguous material. The most often used procedure is text-based scenario training in which participants read ambiguous scenarios that are consistently disambiguated in a positive way by completing word-fragments and answering comprehension questions [6]. The first studies employing this paradigm showed that interpretation biases could be modified in adults, with corresponding effects on emotional vulnerability [6].

In the past decade, research on the possibility to use CBM-I as an intervention to reduce emotional symptoms or stress-reactivity has grown rapidly [7]. Promising findings have been obtained in populations ranging from healthy adolescents [8], [9], to clinically anxious youth [10], [11], but null-findings have also been published [12]. Recent meta-analyses show mixed effects [13], [14], with relatively robust changes in interpretation bias, but small and sometimes non-significant emotional effects. One of the limitations in interpreting the meta-analytic findings is the large heterogeneity in training tasks, number of training sessions, sample types, and assessment methods. Although some moderators have been found, it is difficult to disentangle them, as, for example, the number of sessions (ranging from 1–12 sessions) is often confounded with symptom severity [14]. It is therefore also difficult to decide what might increase CBM-I efficacy, but a recent review suggested imagery as one possibility [15]. While the original scenario paradigm includes an imagery component, CBM-I methods with a greater imagery focus have been developed and applied in the context of depression, with initial studies showing promising effects on interpretations and symptoms of depression [16], [17], [18]. A recent meta-analysis including such procedures found a medium effect for reduction in depressive symptoms when averaged across all control comparisons [19].

Mental imagery, defined as ‘representations and the accompanying experience of sensory information without a direct external stimulus’ [20], plays an important role in both anxiety and depression (for reviews, see [21], [20]). Previous research has shown that processing stimuli using active imagery has stronger effects on interpretation bias and emotional vulnerability than processing the same stimuli verbally [22], [23]. Further, many anxiety disorders are characterized by distressing mental images, like flashbacks to traumatic events [24], or mental images of embarrassment in social anxiety [25], and depression has been associated with difficulties in mental imagery of (future) positive events [26]. Imagery might thus be an important target for treatment.

One imagery-focused CBM-I paradigm is a ‘picture-word’ training, in which ambiguous pictures are paired with positive words and participants have to combine the two to form a positive mental image. This has been found to affect interpretation bias, mood, and behavior in dysphoric adults [27], and has formed part of CBM-I interventions investigated in depressed samples [28], [17]. However, this picture-word training has not yet been studied in comparison to the more traditional scenario training or to a neutral control condition, nor has it been applied in adolescents at risk for anxiety or depression. As text-based scenario training has been reported to be experienced as relatively boring in adults [29] and requires participants to read many lines of text, a more visually based interpretation training might be more attractive for adolescents. A pilot-study suggested efficacy of the picture-word training in increasing positive affect and reducing negative interpretations amongst healthy adolescents boys [30].

To fully appreciate the potential of an intervention in reducing or preventing anxiety or depression, long-term assessments are crucial. Until now, research on long-term effects of text-based scenario training has been limited, and only a couple of studies have included follow-up assessments. In adults, reductions in social anxiety have been observed several weeks after text-based scenario CBM-I [31], [32], although null findings at long-term follow-up have also been reported [33]. A study on a combined training including both text-based scenario CBM-I and attentional bias training for adolescents with heightened social and/or test anxiety symptoms [34], found long-term reductions in interpretation bias at two-year follow-up. Also, a small effect on social anxiety was observed after six months, but this difference between the CBM training and the control group was no longer significant at longer term follow-up [35].

The aim of the current study was to investigate the short- and long-term effects of two types of online interpretation training in adolescents with heightened symptoms of anxiety or depression. Adolescents were selected on symptom level, as research on adults has indicated that CBM-I might be particularly effective in at-risk, subclinical, or clinical samples [14]. Participants were randomized to eight sessions of either a text-based scenario training, a picture-word imagery training, or a neutral text-based scenario control training. Primary outcomes of anxiety and depressive symptoms, and secondary outcomes of self-esteem, perseverative negative thinking, and social-emotional and behavioral problems were assessed pre- and post-training (short-term) and at three and six months follow-up (long-term). Interpretation bias (two tasks) and stress-reactivity were assessed pre- and post-training. We hypothesized that compared to the control group, both scenario and picture-word training would reduce anxiety, depression, and negative interpretation bias, and improve emotional resilience as assessed with secondary emotional outcome measures. To explore for whom training might work best, we investigated potential moderating effects of baseline interpretation bias, and baseline imagery use. We hypothesized that training effects would be larger for those participants with a more negative interpretation bias [36], [37], and, particularly for the imagery-based picture-word training, with a greater tendency to use imagery in daily life [17]. Furthermore, we explored how participants experienced the training and how performance and imagery developed over the course of training.

## Methods

### Participants

Participants were recruited from four secondary schools in the Netherlands in February 2015 and follow-up was completed in November 2015. A power analysis was performed in G*power 3.1 [38] with the following parameters: a small to medium effect size of *f* = .20 (correlation coefficient, based on [17], [14], [39]); an alpha (two-sided) of 0.0056 (Bonferroni Holm correction for 9 outcome measures); a power of .80; three groups; four measurements; a correlation between measurements of .5; and a non-sphericity correction of 0.34. This analysis revealed that a sample size of 150 participants was needed to detect a Condition x Time interaction for our primary outcome measures of anxiety and depressive symptoms. Recruitment stopped when the planned sample size was reached.

**Fig 1:**
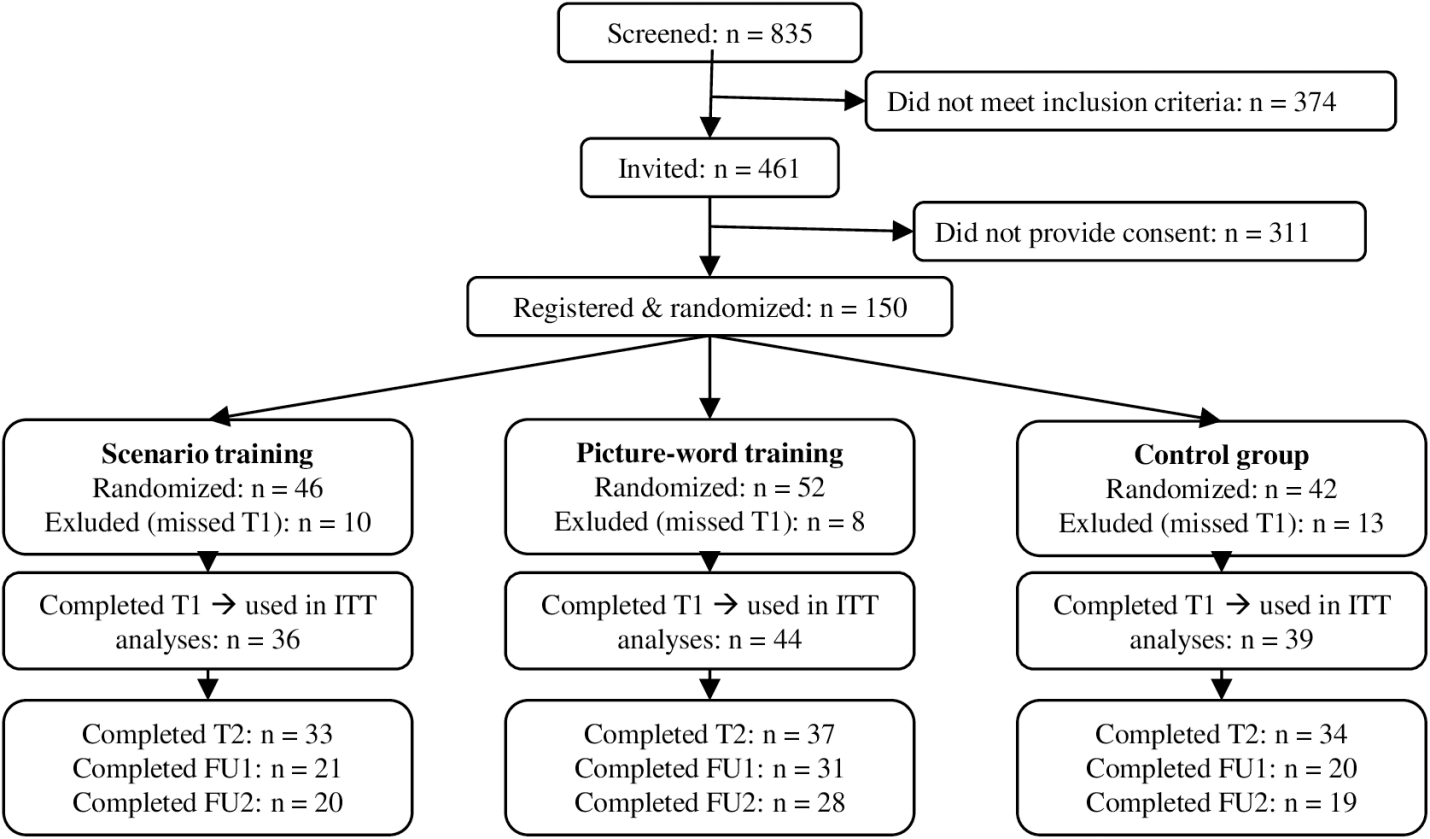
Flow chart. T1 = pre-training assessment; T2 = post-training assessment; FU1 = 3 months follow-up; FU2 = 6 months follow-up; ITT = intention-to-treat.

Inclusion criteria were: scholars in the 1^st^ to 6^th^ grade (aged 11–19) of a regular (all levels except for special education) high school (for screening and training study); a score > 16 on the Screen for Child Anxiety Related Emotional Disorders (SCARED) and/or > 7 on the Children's Depression Inventory (CDI) (training study); and parental consent (passive for screening; active for training study). Cut-off scores were determined based on a previous study by our research group in a sample of 681 unselected adolescents, where 50% of adolescents scored above these values [40]. After screening 835 adolescents for anxiety and depressive symptoms, adolescents scoring above the inclusion cut-offs were invited to take part in the study (*n* = 461). Active informed consent from both the adolescent and a parent was obtained from 150 adolescents and they were randomized across the three parallel conditions (see Fig 1 for a flow diagram). Randomization was stratified by school and gender, and determined by a computerized procedure (1:1:1 ratio) at the point when a participant registered themselves online for the pre-training assessment, thus ensuring allocation concealment. The randomization procedure was written by a programmer independent of the study, and both participants and test assistants were blind to allocation. Participants who missed the first assessment were excluded (*n* = 31). The remaining 119 participants (63% female, mean age 15.68, standard deviation (*SD)* = 1.33) were included in the intention-to-treat analyses (scenario: *n* = 36, picture-word: *n* = 44, control: *n* = 39). The training groups did not differ on demographic characteristics or baseline scores on outcomes measures, all *p’s* > 0.17 (see Tables 1 and 2).

**Table 1:** Demographic characteristics per condition.

	Scenario training (*n* = 36)	Picture-word training (*n* = 44)	Control group (*n* = 39)
Age, mean (*SD*)	15.78 (1.35)	15.76 (1.22)	15.51 (1.43)
Female, N (%)	23 (63.9)	26 (59.1)	26 (66.7)
Sessions completed, mean (*SD*)	5.56(3.05)	5.91(2.61)	6.05(2.65)

**Table 2:** Observed means and standard deviations per condition, and effect sizes for difference in change between conditions.

Outcome measure^a^	Condition	T1 pre-training assessment		T2 post-training assessment		Between-group^c^ ES, T2-T1		FU1 3 months follow-up		Between-group ES, FU1-T1		FU2 6 months follow-up		Between-group ES, FU2-T1	
		*M*	*SD*	*M*	*SD*	*d*	*CI*	*M*	*SD*	*d*	*CI*	*M*	*SD*	*d*	*CI*
SCARED	Scenario	27.41	12.88	24.55	13.64	0.07	-0.31–0.44	25.00	13.09	0.16	-0.23–0.56	21.15	13.75	0.02	-0.40–0.43
	Picture-word	25.25	11.32	22.43	12.47	0.12	-0.25–0.49	20.35	13.04	0.16	-0.24–0.56	18.93	13.35	0.21	-0.21–0.63
	Control	24.82	12.83	22.18	12.44	-	-	22.00	12.79	-	-	20.68	13.98	-	-
CDI	Scenario	13.58	7.95	11.39	9.34	0.37	-0.02–0.75	10.57	8.76	0.27	-0.12–0.66	11.45	8.35	0.22	-0.19–0.64
	Picture-word	13.77	7.11	12.65	8.30	0.12	-0.26–0.49	9.32	5.38	0.23	-0.16–0.62	10.57	6.29	0.16	-0.26–0.58
	Control	11.69	7.33	11.18	6.97	-	-	10.80	9.34	-	-	10.32	9.63	-	-
REC-T	Scenario	-0.28	0.69	-0.73	0.81	0.34	-0.04–0.71	-	-	-	-	-	-	-	-
	Picture-word	-0.47	0.68	-0.41	0.74	0.25	-0.14–0.62	-	-	-	-	-	-	-	-
	Control	-0.22	0.52	-0.39	0.68	-	-	-	-	-	-	-	-	-	-
SST	Scenario	35.01	17.25	33.15	19.11	0.08	-0.31–0.46	-	-	-	-	-	-	-	-
	Picture-word	38.95	20.34	37.64	20.12	0.21	-0.17–0.59	-	-	-	-	-	-	-	-
	Control	39.05	18.77	34.21	17.18	-	-	-	-	-	-	-	-	-	-
Positive mood^b^	Scenario	169.36	65.39	161.88	74.87	0.10	-0.31–0.46	-	-	-	-	-	-	-	-
	Picture-word	165.35	60.14	154.80	71.04	0.03	-0.36–0.41	-	-	-	-	-	-	-	-
	Control	179.09	67.41	165.32	75.90	-	-	-	-	-	-	-	-	-	-
Negative mood^b^	Scenario	66.91	64.77	67.70	68.93	0.03	-0.35–0.41	-	-	-	-	-	-	-	-
	Picture-word	63.92	48.74	70.06	63.71	0.17	-0.22–0.55	-	-	-	-	-	-	-	-
	Control	52.56	50.06	50.00	52.92	-	-	-	-	-	-	-	-	-	-
RSES	Scenario	26.25	4.85	26.88	4.93	0.03	-0.35–0.41	27.90	4.75	0.01	-0.39–0.41	27.45	4.54	0.09	-0.34–0.51
	Picture-word	26.11	5.00	26.81	5.09	0.08	-0.29–0.46	28.00	4.98	0.21	-0.20–0.62	27.96	4.44	0.01	-0.42–0.44
	Control	27.18	5.41	28.21	5.99	-	-	28.77	5.95	-	-	29.05	5.86	-	-
PTQ	Scenario	43.58	13.25	41.21	13.79	0.17	-0.21–0.55	40.95	13.19	0.05	-0.36–0.46	39.40	13.51	0.11	-0.31–0.53
	Picture-word	42.34	12.38	40.00	13.60	0.36	-0.02–0.73	36.71	14.14	0.12	-0.30–0.54	33.89	13.21	0.00	-0.42–0.43
	Control	43.05	12.43	38.24	12.50	-	-	38.30	13.28	-	-	35.84	9.46	-	-
SDQ-P	Scenario	8.29	4.08	8.19	4.42	0.04	-0.35–0.44	6.15	3.96	0.48	0.01–0.96	6.40	3.94	0.03	-0.39–0.46
	Picture-word	6.59	5.20	5.80	3.49	0.03	-0.36–0.42	4.88	4.13	0.05	-0.42–0.52	7.10	5.82	0.50	0.08–0.93
	Control	6.52	4.02	6.19	3.64	-	-	5.68	4.40	-	-	4.95	3.30	-	-

^a^ SCARED = Screen for Child Anxiety Related Emotional Disorders; CDI = Children’s Depression Inventory; REC-T = Recognition Task; SST = Scrambled Sentence Task; RSES = Rosenberg Self-Esteem Scale; PTQ = Perseverative Thinking Questionnaire; SDQ-P = Strengths and Difficulties Questionnaire (Parent)

^b^ Note that for positive and negative mood, T1 and T2 refer to pre- and post-stressor mood respectively, both assessed at the post-training assessment session.

^c^ Compared to the Control group as reference category

### Scenario training (experimental and control)

**Fig 2:**
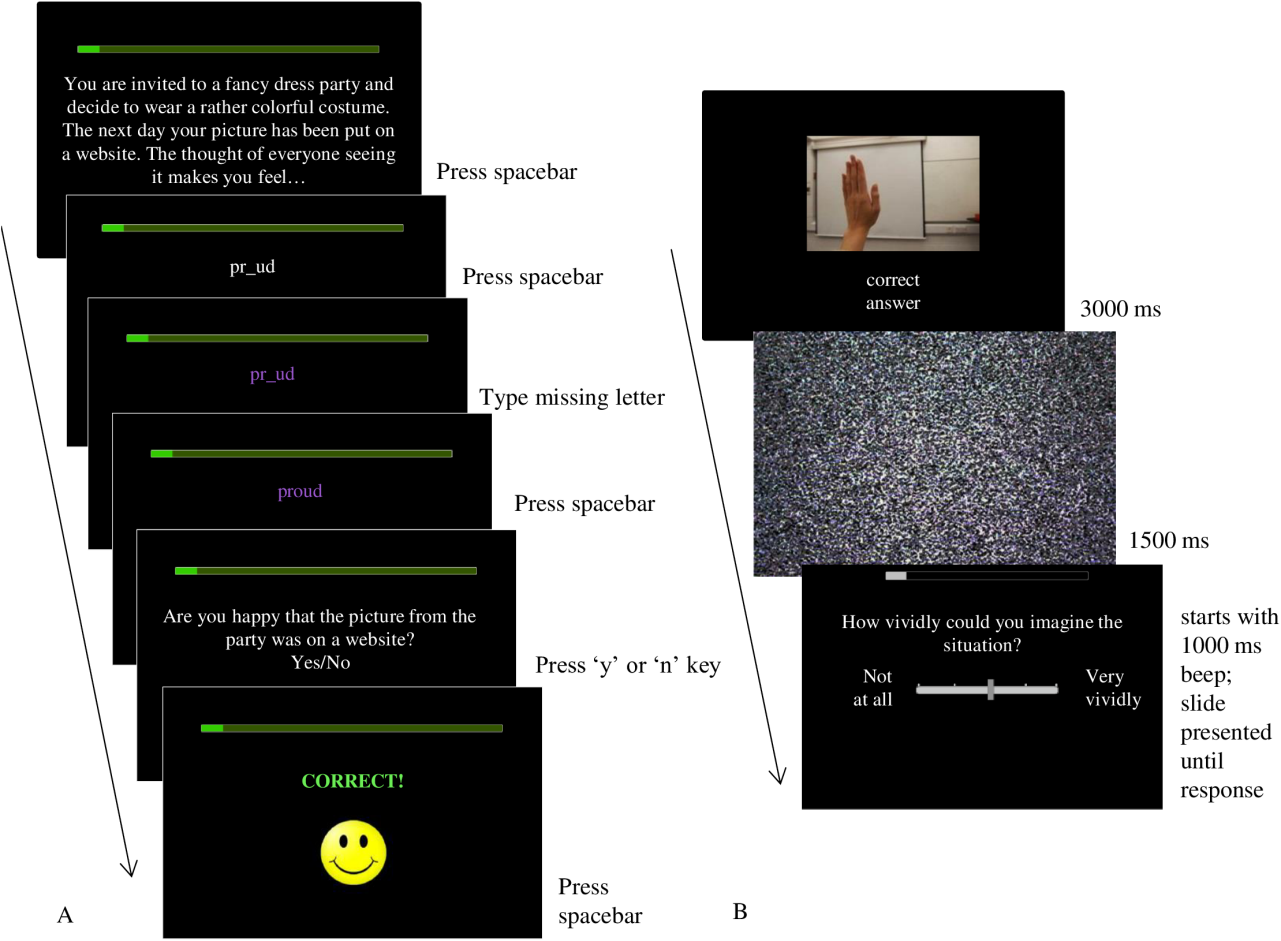
**Example of typical trial in the scenario training (A) and picture-word training (B)**.

The scenario training and control group completed the experimental or a control version, respectively, of the scenario paradigm developed by Mathews and Mackintosh [6]. In this task, participants were presented with 3-line ambiguous scenarios, with a missing word in the last sentence. This word was then presented as a word-fragment, and participants had to press the spacebar as soon as they recognized the word, and complete it by pressing the key corresponding to the first missing letter (see Fig 2A for an example). In the experimental condition, completing the word-fragment disambiguated the training scenarios in a positive way. In the control condition, the scenarios started with the same sentence as the scenarios in the experimental condition, and thus were in the same context, but here, they ended in a neutral way. Each interpretation was reinforced by a comprehension question about the scenario, followed by feedback.

An example (positive) scenario might be: ‘You are invited to a fancy dress party and decide to wear a rather colorful costume. The next day your picture has been put on a website. The thought of everyone seeing it makes you feel pr-ud (proud)’. ‘Are you happy that the picture from the party was on a website? (Yes)’. The neutral version might be: ‘You are invited to a fancy dress party and decide to wear a rather colorful costume. The next day your picture has been put on a website. You show the picture to your si-ter (sister)’. ‘Did someone take a picture of your costume? (Yes)’.

In each training session, three blocks of 14 trials each were presented, which consisted of 10 training scenarios (with positive or neutral resolutions in the experimental or control group respectively) and two positive and two negative probe scenarios (disambiguated in a positive or negative way respectively). Probe scenarios were used to assess changes in interpretation bias during training, with relatively longer RTs in response to positive probes compared to negative probes indicating a negative interpretation bias. The same pre-randomized order of scenarios was applied to all participants.

Participants were asked to imagine the scenarios as vividly as possible and as happening to themselves. After each 4^th^ trial, participants rated to what extent they were able to imagine the outcome of the scenario on a 4-point scale. A progress bar indicated how many trials were left in each block. Between blocks, short breaks were provided with feedback, consisting of the number of points earned based on performance (one point for each correct answer, to word fragments and comprehension questions).

All scenarios for both the experimental and control condition were developed or obtained from other researchers for use in a previous study in Dutch adolescents [40] and included relevant situations from adolescent daily life that could provoke anxiety- or depression related negative interpretations.

### Picture-word training

The picture-word training group received an imagery focused interpretation training based on the picture-word task [30], [41]. In this task, participants were presented with pictures of all kind of situations, representing daily adolescent life (e.g., school, traffic, sports, friends), or some special events (holiday, extreme sports). Pictures could be interpreted in both relatively positive and negative ways. In the training task, all pictures were combined with one or several words that gave a positive interpretation to the situation. For example, a picture of a school exercise was presented together with the words ‘quite easy’. The first training session started with participants reading an extensive introduction consisting of an imagery exercise (cutting a lemon), examples of stimuli, and instructions explaining what imagery is, and how it is possible to imagine everything, even if it would not happen to you in real life. The instructions encouraged participants to imagine what they would see, feel, hear, smell, and taste in each situation. The pictures (640(W) x 480(H) pixels) and word(s) (Arial 30pt) were presented simultaneously for 3000 ms and followed by a scrambled black and white screen for 1500 ms. Participants were asked to close their eyes as soon as they had seen the picture and to imagine the scene as happening to themselves and as vividly as possible. A 1000 ms beep indicated that they could open their eyes again, after which they were asked to rate how vividly they could imagine the scene (1 = not at all and 5 = very vividly). The next trial started immediately after their response (see Fig 2B for an example). The task consisted of six blocks of 10 trials. After each block, participants were asked to what extent they experienced the last scene as happing to themselves and to describe (type in) what they felt, heard, saw, smelled, etc. Before starting the next block, they were reminded of the importance of imagining the scene as happening to themselves, including the aforementioned possible sensory experiences and concentrate on the image rather than their thoughts. During training, a progress bar indicated how many trials were left in each block. Stimuli were partly drawn and translated from previous studies in dysphoric adults [27] and healthy adolescents [30]. New stimuli were developed for the current study as well, representing particularly Dutch scenes. The stimuli were piloted with several students and adolescents.

### Primary outcome measures

Anxiety symptoms were assessed with the Screen for Child Anxiety Related Emotional Disorders (SCARED, [42]), a 41-item (rated 0–2) self-report questionnaire assessing social phobia, separation anxiety, generalized anxiety, panic/somatic symptoms and school phobia.

Depressive symptoms were assessed with the Children’s Depression Inventory (CDI, [43]), a 27-item self-report questionnaire with items consisting of three statements indicating varying levels of depressive symptomatology (rated 0–2).

Internal consistency for the primary outcome measures was good to excellent in the current sample, SCARED *α* = .92, and CDI *α* = .88.

### Secondary cognitive outcome measures

The Recognition Task (REC-T, [6]) was used to assess interpretation bias. Here, ambiguous scenarios were read and completed with word-fragments as in the scenario training, but both the word-fragment and comprehension question did not resolve the ambiguity. After presentation of eight scenarios, titles of these scenarios were randomly presented again, paired with both a negative and a positive interpretation, presented in random order. Participants rated to what extend the interpretation corresponded to the scenarios on a 4-point scale (1 = not at all and 4 = fully). An interpretation bias index was computed by subtracting ratings for positive interpretations from ratings for negative interpretations; a higher score indicated a negative interpretation bias. Two sets were created to use pre- and post-training, and they were counterbalanced across participants.

Interpretation bias was also assessed with a computerized version (based on [44]) of the Scrambled Sentence Task under cognitive load (SST, [45]). In this task, participants were presented with scrambled sentences of six words (for 8000 ms, or until a response was given). They had to unscramble the sentence as quickly as possible into a grammatically correct sentence of five words. They pressed the spacebar as soon as they recognized a sentence and then clicked on the corresponding words in the correct order. A fixation cross was presented at the left side of the screen for 500 ms before the next sentence appeared. All scrambled sentences were self-referent and contained two possible sentences: a positive and a negative one (e.g., ‘good make I impression bad a’). A higher number of negatively resolved sentences indicated a negative interpretation bias. The task consisted of three blocks of 10 trials. At the start of each block, a four-digit number was presented [30], which participants had to report at the end of the block. The aim of this cognitive load was to better tap into implicit interpretation processes by preventing response tendencies. Stimuli were selected and translated from [44] and [30], creating an adolescent-friendly set with sentences reflecting both anxiety and depression relevant statements. Two sets were created to use pre- and post-training, and they were counterbalanced across participants.

### Secondary emotional outcome measures

Stress reactivity was assessed by recording emotional responses to an anagram stress task (cf. [46]). Participants were presented with 15 anagrams that had to be solved within 30 seconds by typing in the correct word. A new anagram was presented after responding or when the 30 seconds were expired. Some of the anagrams were easy to solve, but most were extremely difficult (range from 6–14 letters). Participants were told that the anagrams would be reasonably easy and that performance was related to intelligence. Before and after the stress task, participants rated to what extend they felt sad, nervous, anxious, enthusiastic, happy and relaxed, using visual analogue scales. Scores were combined into a negative and positive mood scale respectively.

Self-esteem was assessed with the Rosenberg Self-Esteem Scale (RSES, [47]), a 10-item (rated 1–4) self-report questionnaire.

The Perseverative Thinking Questionnaire (PTQ, [48]) was used to assess worry and rumination. The PTQ is a 15-item (rated 1–5) self-report questionnaire assessing key features of repetitive negative thinking (repetitive, intrusive and difficult to disengage from) and the unproductiveness of and mental capacity captured by this thinking.

The Strengths and Difficulties Questionnaire parent version (SDQ-P, [49]) is a 25-item (rated 0–2) parent-report questionnaire assessing emotional problems, conduct problems, hyperactivity-inattention and peer problems as well as pro-social behavior. The total difficulties score, computed based on all problem subscales, was used in this study. Internal consistency for the secondary emotional outcome measures was adequate to excellent in the current sample (RSES *α* = .87, PTQ *α* = .95, SDQ-P *α* = .62, positive mood *α* = .79, negative mood *α* = .68).

In order to be able to assess cost-effectiveness, we also included questionnaires on quality of life (EQ-5D-Y, self-report) and health related costs (parent-report), but these data are not included in the current manuscript.

### Daily imagery use

An adaptation of the Spontaneous Use of Imagery Scale (SUIS, [50]) was used to assess spontaneous use of mental imagery in daily life. The original 12-items version was reduced to seven items, which were reformulated to be suitable for Dutch adolescents (based on [51], [52]). Items described daily situations where imagery might be used or come to mind and participants had to indicate how often this would be the case for them (1 = never, and 5 = always). The adapted SUIS was validated in 144 unselected adolescents who participated in one of our previous studies [40], with Cronbach’s alpha = .71. Cronbach’s alpha in the current sample was .72.

### Evaluation questionnaire

An evaluation questionnaire was administered at the post-training assessment, assessing participant experiences with the training. Questions were related to clarity of instructions and aim of the training, enjoyment, difficulty, concentration, learning experiences, satisfaction, and willingness to train again or recommend the training. Participants also read here that there had been a ‘real and ‘fake’ training, and had to indicate in which training condition they thought they had been.

### Procedure

This study was approved by the ethics committee of the psychology department of the University of Amsterdam, carried out in accordance with the World Medical Association Declaration of Helsinki, and registered in the Dutch trial register with number NTR4850 prior to the start of recruitment. Please note that the trial registration contains information about six arms involving 300 participants. Participants were initially recruited from four schools and randomized into three of the arms; in a second phase participants were recruited from a different set of four schools and randomized into the other three arms (the current study). Although the two phases are registered in one registry entry, they are treated as two separate studies as the recruitment, sampling, and randomization were independent of one another. Results from the other three arms (the first phase) are reported elsewhere [53]. During the first phase, but prior to the start of the current phase, inclusion criteria were changed due to limited inclusion: initial cut-off scores were SCARED > 26 and/or CDI > 11 (25% highest scores in our previous study [40]).

Adolescents and parents of participating school classes received an information letter about the screening and could indicate via school or the principal investigator if they did not want to participate (passive consent). The screening was completed under supervision during regular school hours in a computer classroom. Participants scoring above the cut-off were selected by a computerized procedure and those adolescents and their parents received another information letter inviting them for the training study. The aim of the study was explained as ‘investigating a training to make adolescents more resilient to stress and negative emotions, like feeling anxious or down’. When adolescents and their parent provided written informed consent, they were invited for the first assessment (three to five weeks after screening). This pre-training assessment (T1) took place in a computer classroom after the last school lesson (due to scheduling difficulties, for some adolescents, the assessment took place during school hours), in a group of adolescents under supervision of one or two research assistants. Assessment started with an Emotional Visual Search Task (to compare with data of the other arms [53]), the REC-T and the SST, followed by the questionnaires (RSES, SCARED, PTQ, CDI, and EQ-5D-Y, in fixed order), and took approximately one hour. Training was performed online at home during the following four weeks. Participants received eight training sessions of approximately 15 minutes each, which they could complete whenever they wanted, although they were encouraged to complete them within two days. A new session became available twice a week, and was announced by e-mail and text message. Reminder e-mails were sent after two and five days, and participants who had not trained for more than seven days were contacted once by telephone. Technical assistance was offered where necessary. After four weeks, the post-training assessment (T2) took place at school, again after the last lesson. The same procedure as T1 was followed, but here the questionnaires were appended by the anagram stress task. When all participants in a room had finished this task, they were immediately debriefed on the stress task, before they were asked to complete the evaluation questionnaire. Three and six months after T2 (FU1 and FU2), participants received an e-mail and text message to invite them to the follow-up questionnaires, which could be completed online. Reminder e-mails were sent after two weeks, and participants who did not respond within three weeks were contacted by telephone. Parents also received an e-mail to complete their questionnaires at T1, T2, FU1 and FU2 and were sent reminders after one and two weeks. Participants were compensated by vouchers and participation in a lottery, with the amount of compensation based on the number of training and assessment sessions completed (5–15 euro).

### Data analyses

Analysis of variance (ANOVA) was used to explore potential differences between training groups in age, daily imagery, baseline scores on all outcome measures, and number of training sessions completed. Chi-square tests were used to compare gender, completion rates of assessments, and responses to the evaluation questionnaire. Bivariate Pearson’s correlation coefficients were computed to assess the relations between emotional and cognitive outcome measures, and baseline and in-training imagery- and vividness scores (see Table 3). Independent sample t-tests were used to test potential differences between the scenario and control group in accuracy and imagery during training.

**Table 3:** Correlations at baseline.

	SST	SCARED	CDI	RSES	PTQ	SDQ-P	SUIS
REC-T ^a^	.51***	.48***	.46***	-.41***	.38***	.21*	.20*
SST		.62***	.75***	-.70***	.65***	.11	.22*
SCARED			.68***	-.57***	.62***	.18	.31**
CDI				-.77***	.68***	.14	.19*
RSES					-.64***	-.14	-.09
PTQ						.07	.24**
SDQ-P							.01

^a^ REC-T = Recognition Task; SST = Scrambled Sentence Task; SCARED = Screen for Child Anxiety Related Emotional Disorders; CDI = Children’s Depression Inventory; RSES = Rosenberg Self-Esteem Scale; PTQ = Perseverative Thinking Questionnaire; SDQ-P = Strengths and Difficulties Questionnaire (Parent); SUIS = Spontaneous Use of Imagery Scale

* *p* < 0.05

** *p* < 0.01

*** *p* < 0.001

To assess potential training effects, mixed regression analyses were performed, as this method is suitable to deal with multiple assessments within participants and uses all available data without discarding participants with missing data [54], [55]. For all outcomes measures, a mixed model with participants as grouping variable and Time as a repeated measures variable was tested using maximum likelihood estimation. This model includes random intercepts at the participant level. With regard to the covariance between time points, we verified (based on AIC and BIC criteria) whether these were structured according to compound symmetry, or first order autoregressive, or whether these were unstructured. The factor Time had two levels for REC-T, SST and mood scales (T1 and T2), four levels for SCARED, CDI, RSES, PTQ, and SDQ-P (T1, T2, FU1, and FU2), and eight levels for training performance measures (one for each training session).

To test our hypotheses regarding training effects, separate models were created for all outcomes measures including the fixed factors Condition and Time, and their interaction. The best model was selected in a backward elimination procedure, in which parameters were excluded from the model based on AIC and BIC criteria and significance level of the parameters. Next, for the primary outcomes measures, baseline interpretation bias and baseline imagery were tested for their potential moderating role by separate models including these variables, Condition, Time, and all possible interactions, and again excluding parameters till the best model was obtained. Changes in RTs to probe scenarios were analyzed for the scenario and control training group only in the same fashion, starting with a model including Condition, Time, and their interaction. For imagery ratings during picture-word training, a model including only Time as a linear predictor was created, as no other conditions were involved and Time as a linear predictor resulted in a better fit than Time as a factorial predictor.

We also explored whether potential training effects on our primary outcomes measures were influenced by the condition participants thought they were in (experimental or control), or by the imagery ratings (scenario training and control) or vividness ratings (picture-word training) during training, by including all interaction terms including these variables in separate models.

Effect sizes were calculated for the between-group differences in change from T1 to T2, FU1, and FU2, comparing both the scenario and picture-word group to the control group (see Table 2). The t-values and degrees of freedom of the relevant fixed effects estimates derived from the mixed models were used to calculate Cohen’s d, with the d = 2t/(sqrt(df) formula. Estimated parameters from the mixed models were also used to calculate CIs for the effect sizes, following [56].

Bonferroni-Holm correction was applied to control for Type I errors related to the number of outcome measures, and adjusted p-values are reported. Effects with uncorrected *p* <0.05 that lost significance after correction were defined as marginal. Statistics of the original and final models for all hypotheses can be found in Tables 4 and 5. Table 6 shows the relevant parameters estimates (with T1 and the control group as reference categories).

**Table 4:** Statistics of the original and final models (part 1).

Outcome measure^a^	Model ^b^	Model fit		Time		Condition		Condition x Time		Time x Moderator		Condition x Time x Moderator	
		*AIC*	*BIC*	*F*	*df*	*F*	*df*	*F*	*df*	*F*	*df*	*F*	*df*
SCARED	Condition x Time (UN)	2508.66	2594.34	14.46***	3,81.90	0.52	2,117.58	0.56	6,81.74	-	-	-	-
	**Time (UN)**	2497.05	2551.57	14.28***	3,81.16	-	-	-	-	-	-	-	-
	Condition x Time (UN) x Baseline REC-T	2488.18	2620.59	13.63***	3,82.97	1.16	2,117.27	0.48	6,82.60	1.12	3,79.49	1.95	6,79.77
	Condition x Time (UN) x Baseline SST	2339.01	2469.79	16.15***	3,79.37	2.66	2,111.14	0.40	6,79.26	0.34	3,77.51	1.60	6,77.38
	Condition x Time (UN) x Baseline SUIS	2504.01	2636.42	14.25***	3,83.85	1.01	2,120.07	0.65	6,83.94	0.90	3.88.62	2.02^†^	6,88.84
	Condition x Time (UN) x Imagery (ST /CTR)	1450.83	1537.36	2.68	3,45.23	0.00	1,67.63	2.61	3,45.23	1.77	3,44.43	3.03^†^	3,44.43
	Time (UN) x Vividness (PWT)	943.96	996.78	2.33	3,31.25	-	-	-	-	2.08	3,30.79	-	-
CDI	Condition x Time (UN)	2166.11	2251.73	11.55***	3,79.62	0.33	2,118.93	0.80	6,79.49	-	-	-	-
	**Time (UN)**	2156.03	2210.51	11.77***	3,79.16	-	-	-	-	-	-	-	-
	Condition x Time (UN) x Baseline REC-T	2137.66	2269.98	13.93***	3,79.79	1.49	2,119.47	1.07	6,79.52	5.60*	3,78.25	1.37	6,78.37
	**Time (UN) x Baseline REC-T**	2125.77	2195.82	13.14***	3,78.31	-	-	-	-	4.60*	3,77.54	-	-
	Condition x Time (UN) x Baseline SST	1968.77	2099.45	12.97***	3,74.74	0.77	2,109.25	1.20	6,74.76	2.80^†^	3,75.11	2.21	6,75.08
	Condition x Time (UN) x Baseline SUIS	2166.15	2298.46	10.05***	3,80.72	0.65	2,122.62	1.07	6,80.38	1.84	3,83.47	2.23^†^	6,83.20
	Condition x Time (UN) x Imagery (ST/CTR)	1258.88	1345.28	1.72	3,42.88	0.07	1,67.95	0.52	3,42.88	1.19	3,42.89	0.67	3,42.89
	Time (UN) x Vividness (PWT)	815.81	868.63	1.62	3,32.41	-	-	-	-	1.12	3,32.08	-	-
REC-T	**Condition x Time (UN)**	454.32	485.06	7.75*	1,109.48	1.28	2,117.44	4.62	2,109.49	-	-	-	-
SST	Condition x Time (UN)	1821.79	1852.21	3.00	1,103.85	0.80	2,118.03	0.59	2,103.84	-	-	-	-
	**Time (CS)**	1814.52	1828.04	2.84	1,103.79	-	-	-	-	-	-	-	-

^†^*p* < 0.10

* *p* <0.05

** *p* <0.01

*** *p* <0.001, after Bonferroni-Holm correction.

^a^ SCARED = Screen for Child Anxiety Related Emotional Disorders; CDI = Children’s Depression Inventory; REC-T = Recognition Task; SST = Scrambled Sentence Task; SUIS = Spontaneous Use of Imagery Scale; PWT = Picture Word Training; ST = Scenario Training; CTR = Control group; UN = Unstructured covariances; CS = Compound Symmetry covariance structure

^b^ Bold print = final model (note that moderation models were tested after testing general training effects on primary outcomes measures (SCARED and CDI)).

**Table 5:** Statistics of the original and final models (part 2).

Outcome measure^a^	Model ^b^	Model fit		Time		Condition		Condition x Time	
		*AIC*	*BIC*	*F*	*df*	*F*	*df*	*F*	*df*
Positive mood	Condition x Time (UN)	2268.82	2298.77	4.58	1,102.23	0.42	2,105.56	0.13	2,102.23
	**Time (UN)**	2262.01	2278.65	4.57	1,102.17	-	-	-	-
Negative mood	Condition x Time (UN)	2217.41	2247.36	0.35	1,102.11	1.15	2,104.68	0.40	2,102.10
	**Time (UN)**	2212.23	2228.87	0.37	1,102.01	-	-	-	-
RSES	Condition x Time (UN)	1975.32	2061.11	9.74***	3,85.49	0.80	2,114.32	0.41	6,85.01
	**Time (UN)**	1962.85	2017.45	9.10***	3,84.87	-	-	-	-
PTQ	Condition x Time (UN)	2614.59	2700.20	12.89***	3,86.01	0.14	2,115.82	0.85	6,85.94
	**Time (UN)**	2603.99	2658.47	13.19***	3,86.69	-	-	-	-
SDQ-P	Condition x Time (CS)	1761.12	1815.25	6.63***	3,241.45	1.26	2,114.58	2.55	6,241.48
Probe Bias (ST/CTR)	Condition x Time (UN)	6196.93	6408.73	9.10***	7,57.06	14.40***	1,66.33	0.48	7,57.06
	**Condition + Time (UN)**	6186.14	6369.43	9.09***	7,57.36	24.65***	1,45.35	-	-
PP (ST/CTR)	Condition x Time (UN)	6311.74	6523.54	27.44***	7,53.62	6.00*	1,68.23	0.38	7,53.62
	**Condition + Time (UN)**	6300.36	6483.64	27.60***	7,53.57	6.98*	1,65.36	-	-
NP (ST/CTR)	Condition x Time (UN)	6299.33	6511.13	22.38***	7,37.99	2.25	1,67.45	1.20	7,37.99
	**Time (UN)**	6292.61	6471.83	22.40***	7,37.59	-	-	-	-
Vividness (PWT only)	**Time (UN)**	235.04	369.90	1.87	1,26.30	-	-	-	-

^†^ *p* < 0.10

* *p* <0.05

** *p* <0.01

*** *p* <0.001, after Bonferroni-Holm correction.

^a^ RSES = Rosenberg Self-Esteem Scale; PTQ = Perseverative Thinking Questionnaire; SDQ-P = Strengths and Difficulties Questionnaire (parent); PP = Positive probe; NP = Negative probe; PWT = Picture Word Training; ST = Scenario Training; CTR = Control group; UN = Unstructured covariances; CS = Compound Symmetry covariance structure

^b^ Bold print = final model

**Table 6:** Parameter estimates.

			Scenario training ^a^		Picture-word training		T2			FU1		FU2		T2 Scenario training		T2 Picture word task		FU1 scenario training		FU2 picture-word training	
Outcome measure^b^	Model		*B*	*SE*	*B*	*SE*	*B*	*SE*		*B*	*SE*	*B*	*SE*	*B*	*SE*	*B*	*SE*	*B*	*SE*	*B*	*SE*
SCARED	Time		-	-	-	-	-2.99***	0.56		-4.68***	0.80	-5.02***	0.91	-	-	-	-	-	-	-	-
CDI	Time		-	-	-	-	-1.46***	0.40		-3.03***	0.56	-2.03**	0.60	-	-	-	-	-	-	-	-
REC-T	Condition * Time		-0.06	0.14	-0.25	0.14	-0.16	0.12		-	-	-	-	-0.30	0.17	0.21	0.17	-	-	-	-
SST	Time		-	-	-	-	-2.23^†^	1.33		-	-	-	-	-	-	-	-	-	-	-	-
Positive mood	Time		-	-	-	-	-11.18*	5.23		-	-	-	-	-	-	-	-	-	-	-	-
RSES	Time		-	-	-	-	0.91*	0.30		2.09***	0.40	1.57***	0.41	-	-	-	-	-	-	-	-
PTQ	Time		-	-	-	-	-2.71**	0.76		-5.59***	0.94	-6.09***	1.11	-	-	-	-	-	-	-	-
SDQ-P	Condition * Time		1.92	1.05	0.26	0.99	-0.26	0.45		-0.74	0.51	-1.51*	0.52	-	-	-	-	-1.43	0.75	1.75	0.70
Probe bias ^c^	Condition + Time		-98.63***	19,86	-	-	**-**	-		-	**-**	-	-	-	-	-	-	**-**	-	-	**-**
*Moderation effects*			T2 * REC-T		FU1 * REC-T		FU2 * REC-T			-	**-**	-	-	-	-	-	-	**-**	-	-	**-**
			*B*	*SE*	*B*	*SE*	*B*		*SE*	-	**-**	-	-	-	-	-	-	**-**	-	-	**-**
CDI		Time * REC-T	-0.10	0.64	-1.93*	0.88	-0.53		0.96	-	**-**	-	-	-	-	-	-	**-**	-	-	**-**

^†^ *p* < 0.10

* *p* <0.05

** *p* <0.01

*** *p* <0.001, after Bonferroni-Holm correction.

^a^ Reference categories for parameters estimates were the control condition and pre-training assessment (T1). REC-T was a continuous variable: higher values indicate a more negative interpretation bias. T2 = post-training assessment; FU1 = 3 months follow-up; FU2 = 6 months follow-up

^b^ SCARED = Screen for Child Anxiety Related Emotional Disorders; CDI = Children’s Depression Inventory; REC-T = Recognition Task;; SST = Scrambled Sentence Task; RSES = Rosenberg Self-Esteem Scale; PTQ = Perseverative Thinking Questionnaire; SDQ-P = Strengths and Difficulties Questionnaire (Parent)

^c^ Time effects are not included in this Table, since this model included the eight training sessions as time points. Bias index was significantly reduced at all sessions compared to the first session (all *p’s* < 0.001, parameter estimates between *B* = -416.71, *SE* = 70.41 and *B* = -289.00, SE = 63.99), except for the fourth session, *B* = -47.59, *SE* = 65.53, adjusted *p* = 0.470.

## Results

### Preliminary analyses

Significant correlations were found between both interpretation bias measures, and between these measures and anxiety, depression, self-esteem, perseverative negative thinking, and social-emotional and behavioral problems (see Table 3). Mean levels of anxiety (*M* = 25.8, *SD* = 12.15) and depressive symptoms (*M* = 13.0, *SD* = 7.44) in our sample were around or just below often employed cut-offs for clinical problems (SCARED > 25, [42]; CDI > 16, [57]).

On average, participants completed 5.85 sessions (*SD* = 2.75), and groups did not differ in the number of sessions completed, *F* (2,116) = 0.32, *p* = 0.73. Missing data rates for adolescent data (questionnaires and computer tasks) were 0.8% at T1, 9.4% at T2, 38.7% at FU1, and 43.7% at FU2. Missing data rates for parent-report questionnaires were 13.4% at T1, 13.4% at T2, 42% at FU1, and 34.5% at FU2. Groups did not differ in completion rates at any of these assessment points, all *p’s* > 0.27.

### Primary outcome measures

The hypothesis that anxiety and depression would be reduced by scenario and picture-word training compared to control was not confirmed, as no significant Condition x Time interactions were observed, both adjusted *p’s* > 0.99. For both SCARED and CDI scores, only a significant main effect of Time was found, both adjusted *p’s* < 0.001, indicating significant reductions in symptoms between T1 and all other time points (see Table 6).

Contrary to our hypotheses, training effects on anxiety and depressive symptoms were not moderated by baseline interpretation bias or imagery tendency, as no three-way interactions were observed. However, a significant Time x baseline REC-T interaction effect was found for depressive symptoms, adjusted *p* = 0.012, such that irrespective of training condition, a larger reduction in symptoms was found for those participants who displayed a more negative interpretation bias at baseline (see Table 6).

### Secondary cognitive outcome measures

Our hypothesis that negative interpretation bias would be reduced in the scenario and picture-word training group compared to control was partly confirmed. For REC-T scores, a marginally significant Condition x Time interaction was observed, adjusted *p* = 0.108, indicating a non-significant reduction in interpretation bias in the scenario training group compared to control, adjusted *p* = 0.166 (see Table 6). For SST scores, no significant main effects of Time or Condition, nor interactions were observed, all adjusted *p’s* > 0.19.

### Secondary emotional outcome measures

With regard to stress-reactivity, the expected training effects were not observed, as no significant Condition x Time interactions were observed for positive or negative mood, both adjusted *p’s* > 0.99. For positive mood only, a marginally significant main effect of Time was found, adjusted *p* = 0.105, indicating a decrease in positive mood in response to the stress-task.

For self-esteem and perseverative negative thinking, the expected Condition x Time interactions were also not observed, both adjusted *p’s* > 0.99. Significant main effects of Time were found, both adjusted *p’s* < 0.001, indicating a general increase in self-esteem and reduction in perseverative negative thinking over time.

For SDQ-P, the hypothesized Condition x Time interaction was observed, although only marginally significant, adjusted *p* = 0.160, indicating a marginally significant reduction in parent-reported social-emotional and behavioral symptoms in the scenario training group at FU1 compared to the control group, adjusted *p* = 0.168 (see Table 6).

### Evaluation questionnaire

Responses to the evaluation questionnaire are shown in Table 7. Groups did not differ on the evaluation questions (all *p’s* > 0.25), except for marginally significant differences on the clarity of instructions, χ² (4) = 12.22, adjusted *p* = 0.160, and the ability to concentrate on the training, χ² (4) = 9.95, adjusted *p* = 0.369, with picture-word training participants experiencing instructions as less clear and having more concentration difficulties. Irrespective of condition, most participants (77.7%) thought they were in the control condition. The condition participants thought they were in did not affect any of the results reported above (although a marginal Condition x Time x Perception interaction was observed for PTQ scores, *F* (6, 96.90) = 3.16, adjusted *p* = 0.063, follow-up analyses did not reveal any significant differences between conditions).

**Table 7:** Response to evaluation questionnaire per condition.

		Scenario training	Picture-word training	Control group
The aim of the training was clear before I started	% Agree	39.4	51.4	44.1
	% Not agree	27.3	32.4	26.5
	% Neutral	33.3	16.2	29.4
I enjoyed the training	% Agree	21.2	10.8	17.6
	% Not agree	39.4	62.2	44.1
	% Neutral	39.4	27.0	38.2
The training task was easy^1^	% Agree	84.8	78.4	73.5
	% Not agree	0.0	2.7	5.9
	% Neutral	15.2	18.9	20.6
The instructions on what to do in the training were clear	% Agree	97.0	67.6	88.2
	% Not agree	3.0	21.6	5.9
	% Neutral	0.0	10.8	5.9
I could easily concentrate on the training	% Agree	51.5	24.3	50.0
	% Not agree	30.3	45.9	17.6
	% Neutral	18.2	29.7	32.4
I think I learned to better cope with negative emotions and stress	% Agree	12.1	13.9	14.7
	% Not agree	57.6	61.1	61.8
	% Neutral	30.3	25.0	23.5
I am satisfied with the training	% Agree	75.8	56.8	64.7
	% Not agree	24.2	43.2	35.3
I would recommend the training to a friend who feels anxious or sad	% Agree	24.2	30.6	17.6
	% Not agree	75.8	69.4	82.4
I would train again if I needed help with negative feelings	% Agree	18.2	25.0	11.8
	% Not agree	81.8	75.0	88.2
Which version of the training do you think you got; the 'real' or 'fake' one?	‘Real’	21.2	22.2	23.5
	‘Fake’	78.8	77.8	76.5

^1^Recoded from 'The training task was difficult'. Note that some other questions were included in the questionnaire to evaluate the reward system and the intensity of the training. These data are not included in the current manuscript for reasons of conciseness, but can be requested from the first author.

* *p* < 0.05

### Training performance & imagery

For the scenario training and control group, we assessed interpretation bias based on RTs to probe scenarios during training. For interpretation bias index (RT positive probes–RT negative probes), significant main effects of both Condition and Time were observed, both *p’s* < 0.001, but the expected Condition x Time interaction was not significant, *p* = 0.84. Separate models for RTs to positive and negative probes respectively, revealed that participants became faster in responding to all probes over time, with a larger reduction in RTs to positive probes (hence, a reduction in bias). Furthermore, irrespective of training session, participants in the scenario training group responded faster to positive probes than the control group, adjusted *p* = 0.020. No significant differences were observed between the scenario training and control group in accuracy to word fragments (93.5%) or comprehension questions (92.1%), *p* = 0.42 and *p* = 0.69 respectively, or in imagery ratings during training (*M* = 2.72, *SD* = .044), *p* = 0.79.

For the picture-word training, we explored changes in vividness ratings during training. No significant effect of Time was observed, *p* = 0.18, indicating that vividness ratings (*M* = 3.32, *SD* = .72) did not change over training sessions. The average imagery ratings, which were slightly closer to a very vivid image (5) than to no vivid image at all (1), indicate that in general participants were able to imagine the scenes, but not very vividly.

Baseline imagery use as assessed with the SUIS was not related to imagery ratings in the scenario training and control group, *r* = .00, *p* = 0.98, or to vividness ratings in the picture-word training group, *r* = .15, *p* = 0.33. In the picture-word training group, vividness ratings were significantly correlated with baseline interpretation bias on the SST (*r* = -.40, *p* = 0.010), anxiety (*r* = -.368, *p* = 0.018), depression (*r* = -.41, *p* = 0.006), self-esteem (*r* = .40, *p* = 0.008), and social-emotional and behavioral problems (*r* = -.33, *p* = 0.040), such that participants with less negative bias, fewer symptoms and higher self-esteem gave higher vividness ratings. No such correlations were observed with imagery ratings in the scenario training and control group.

For both types of training, imagery or vividness ratings during training did not affect training effects on interpretation bias or anxiety or depressive symptoms, as no significant interactions with Condition and/or Time were observed, all adjusted *p’s* > 0.16.

## Discussion

The current study investigated the short- and long-term effects of two types of online CBM-I (scenario and picture-word training) on anxiety and depression, negative interpretation bias, and emotional resilience in adolescents with heightened symptoms of anxiety or depression. Contrary to our expectations, no differential training effects were observed on the primary outcome measures of anxiety and depression or on any of the other self-report emotional measures. Irrespective of condition, anxiety and depressive symptoms as well as perseverative negative thinking decreased and self-esteem increased. Emotional responses to the stress task also did not vary between training groups. The stress task only resulted in a marginal overall decrease of positive mood, questioning the credibility of the cover story for our participants. On the only parent-report measure, assessing social-emotional and behavioral problems, the scenario training group showed a faster reduction in symptoms than the control group, and the picture-word training group showed no change at all, but these marginally significant differences at three months disappeared at six months follow-up. To explore for whom training might work best, potential moderators were tested. However, baseline interpretation bias or imagery use did not affect the effectiveness of the experimental trainings compared to control. Overall, neither the scenario nor the picture word training outperformed the control group in terms of emotional effects.

For emotional effects to occur, a change in the targeted cognitive process seems a prerequisite (cf. [58]). Such a change in interpretation bias was only partially present, and solely in the scenario training. Marginally significant group differences were observed on the recognition task, indicating a reduction in negative interpretation bias in the scenario training group compared to the neutral control group. Note that this assessment task closely matched the training task, and effects did not generalize to the scrambled sentence task (another interpretive bias task), which suggests that improvements might have been mainly task-specific. Transfer to other interpretation bias tasks has been hard to find in previous studies as well (e.g., [59], [60]), and the extent to which trained interpretive bias might generalize to other contexts is thus still largely unknown. In the picture-word training group, for which there was no closely-matched bias assessment, the hypothesized change in interpretation bias was not observed.

The overall improvement in terms of anxiety and depressive symptoms and secondary emotional measures resembles the pattern of a previous study in adolescents [35]. These findings might suggest potential ceiling effects, although there seems to be enough room for improvement in adolescents with heightened symptoms. As on average, participants displayed a positive interpretation bias on the recognition task pre-training, one might wonder whether interpretation bias training was indicated for this sample. However, as moderate correlations between the recognition task, the scrambled sentence task (on which participants displayed a negative bias) and anxiety and depressive symptoms were observed, and no absolute cut-offs exist for the recognition task, our sample seemed relatively impaired in making positive interpretations. Still, the scenario training did not have any effects on emotional functioning over and above the control training.

The observed reduction in symptoms in response to neutral control training might indicate that this control training also had a therapeutic effect for our sample of selected adolescents. Exploratory analyses comparing the three current groups (scenario, picture-word, and control) with a matched no-training control group (test-retest condition) from a parallel study [53], revealed short-term (post-training) emotional effects of all three groups, including control, compared to no training. However, all four groups showed comparable reductions in symptoms at follow-up. As the scenarios involved some emotional ambiguity from the start, a neutral ending of the scenario might actually represent a more benign interpretation of the scenario than the interpretation of an emotionally vulnerable adolescent. Thus the control training might have inadvertently been a mild CBM-I training, as it included exposure to emotionally ambiguous information (c.f. [61]), but without confirming pre-existing negative biases in interpretation (cf. [33]). Alternative control conditions could consist of 50/50 positive and negative resolutions [58] or completely neutral scenarios [62], but the first might also comprise a mild training, while the latter would not control for exposure to ambiguous emotional information. Developing a credible control version of CBM-I without unintended training effects remains a challenge for future research (see also [28]). Another potential explanation of reduced symptoms across conditions is regression to the mean, as we pre-selected participants on heightened levels of anxiety and depressive symptoms.

Given the important role of imagery and the potential attractiveness of a more visual training paradigm, the picture-word interpretation training was predicted to be effective in changing interpretations and emotions. However, no training effects on any of the outcome measures were observed following picture-word training compared to the control group. This might be partly explained by difficulties with imagining the positive scenes. While adolescents with heightened symptoms reported more daily use of imagery, they were less able to vividly imagine the positive situations during training (cf. [26]), which might have undermined the potential to change interpretations. Note that most previous studies employing the picture-word paradigm were in adults, and included at least one session that was performed under supervision (e.g., [28] [17]), such that test-assistants could ensure a correct understanding of the imagery procedure. In our study, participants had to read all the instructions at home, and had to type in their sensory experiences once every ten trials. These procedures might have reduced the extent to which participants engaged in the training in the required manner, and also unintentionally reinforced verbal processing. As some of the cognitive processes underlying mental imagery are still developing in children and adolescents [63], more support than simply reading instructions may be needed for adolescents to successfully perform imagery-based interpretation training (e.g., pre-training imagery exercise, cf. [28]). The evaluation questionnaire indeed suggested that picture-word training might have been harder for some participants than the other training paradigms. Although a marginally significant difference was found only for the clarity of instructions and the ability to concentrate on the task, qualitative inspection of the other scores revealed a more negative experience in general (e.g., less enjoyable). Given that previous adult studies have not found this to be the case (e.g., [28]), some aspect of the operationalization in this study might have been suboptimal for facilitating engagement. Finally, neither of the interpretation bias measures used were closely-related to the picture-word training, unlike in other studies that have included an ambiguous picture-rating task (e.g., [27]). Thus, to show any effect on the interpretation measures would have required some degree of transfer, in contrast to the scenario-based training, for which a closely-matched bias assessment was included.

With regard to users’ experience, results further revealed that most participants thought they were in the control condition, as is usual in CBM studies (e.g., [64], [33]), with no difference across training groups. Participants generally would not recommend the training to friends or train again in case of emotional problems. Based on answers to open-ended questions, this seemed to be due to the repetitiveness and unclear rationale of the training (see also [29], [12]), and this, combined with the belief of receiving the control training, might have lowered participants’ motivation to train and reduced efficacy of the training (cf. [29]). If participants simply “go through the motions” of completing the training sessions without actively engaging with the content, we would not expect to see any benefits. Thus, facilitating engagement across multiple training sessions remains a challenge for successful implementation of CBM when delivered remotely, perhaps particularly amongst adolescents who may prefer to spend their time after school otherwise occupied.

Additionally, a limitation of our study is the high drop-out rate at follow-up, which considerably reduced our power to observe training effects. Also note that power was already reduced by the fact that only 119 of the 150 recruited adolescents actually took part in the study. Although online questionnaires seem attractive given their 24/7 accessibility and easy logistics, performing follow-up assessments at schools might be necessary to improve response rates of adolescents. However, assessments at schools also have their drawbacks: the group format used in this study sometimes resulted in noise and distraction, which might have compromised reliability of measurements. Great care should be taken to provide a quiet environment, and equally important, enough privacy to complete tasks and questionnaires.

To summarize, in adolescents with heightened symptoms of anxiety or depression, for both scenario and picture-word training, no significant effects on any of the emotional outcomes measures were observed compared to the control group. A general decrease in symptoms and increase in self-esteem over time was found, consistent with a previous long-term study in adolescents [35]. The scenario training marginally reduced negative interpretation bias, but no such effect was found for the picture-word training (albeit in the absence of a closely-matched bias assessment). Given the absence of emotional effects and the relatively negative evaluation of the training paradigms by participating adolescents, interpretation training as implemented in the current study (i.e. multi-session online training at home, without supervision) should be improved considerably before it could be of practical use for prevention or early intervention. Therefore, a step back from (large-scale) RCTs on effectiveness to experimental research on increasing efficacy of the most promising paradigms and understanding mechanisms of change seems necessary. The challenge for future research is to develop methods to enable the effects of CBM-I observed under controlled laboratory settings to be successfully and robustly transferred to real-world applications.

## Supporting information

S1 File Appendix. Additional measures.
(DOCX)

S2 File CONSORT checklist. (DOCX)

S3 File Trial protocol. S3a, b, and c comprise the complete, original proposal that was reviewed and approved by the ethical committee of the University of Amsterdam. S3d provides the English translation of these documents.
(PDF)
