# Supplementary material for: Imagine the bright side of life: A randomized controlled trial of two types of interpretation bias modification procedure targeting adolescent anxiety and depression
Source: PLoS One. 2017 Jul 17;12(7):e0181147. doi: 10.1371/journal.pone.0181147 (PMC5513454; doi:10.1371/journal.pone.0181147)
Supplement: S1 File — Additional measures. (DOCX) [file pone.0181147.s001.docx]

**Supplement 1: Additional materials**

To assess attentional bias, the Emotional Visual Search Task (EVST, [1]) was used. This task largely resembled a visual search attentional training task ([1], based on [2]). The task consisted of two blocks of 36 trials, where participants had to repeatedly select (by mouse-click) either the only happy face in a 4 x 4 grid of negative faces or the only face with a negative emotion (angry, fearful or sad) in a grid of happy faces. Faces were presented until a response was given and a new trial started once the participants moved the mouse cursor over a fixation cross in the center of the screen. In case of an erroneous response the trial was repeated after feedback. The order of positive or negative blocks was counterbalanced over participants. Face stimuli (height 149, width 117 pixels) were randomly drawn from two sets (counterbalanced over participants) of 36 adolescent faces (18 happy, six fearful, six angry and six sad faces) from the NIMH Child Emotional Faces Picture Set (NIMH_ChEFS, [3], for stimuli selection, see [1]). An attentional bias index was computed by subtracting the average RT for selecting negative faces from the average RT for selecting positive faces.

The EQ-5D-Y [4], a child-friendly version of the EQ-5D [5] was used to assess health-related quality of life at all assessment points. Here, adolescents were asked to rate to what extent they currently experience impairments (1 = no, 2 = (a) little, 3 = very/much) with regard to movement, taking care of themselves, daily activities, pain or other complaints, or negative emotions, and to rate their current health on a scale from 1 to 100.

To assess health-related costs, a parent-report questionnaire was developed for the current study based on the Trimbos /iMTA Questionnaire for Costs associated with

Psychiatric Illness (TiC-P, [6], [7]). Questions were related to the frequency of health care use (general practioner, mental health care institutions, paramedical specialists etc.), use of medicines, and absenteeism from school or other activities. Parents were asked to report on health-related costs retrospectively over the past three months at baseline (T1), and three and six months after completion of the training period (FU1 and FU2).

The full evaluation questionnaire consisted of 22 questions, of which some were based on the Client Satisfaction Questionnaire [8] and some were developed for the current study specifically. Response options varied per question type: yes/no, 4 to 5 answer options, or a 7-point Likert scale. For the analyses reported in the current manuscript, answers were recoded, such that a positive, negative, and neutral (in case of 7 point Likert scales) answer category remained.

**References**

[1] De Voogd EL, Wiers RW, Prins PJM, Salemink E. Visual search attentional bias modification reduced social phobia in adolescents. J Behav Ther Exp Psychiatry. 2014; 45, 252-259. doi: [10.1016/j.jbtep.2013.11.006](http://dx.doi.org/10.1016/j.jbtep.2013.11.006)

[2] Dandeneau SD, Baldwin MW, Baccus JR, Sakellaropoulo M, Pruessner JC. (2007). Cutting stress of the pass: Reducing vigilance and responsiveness to social threat by manipulating attention. J Pers Soc Psychol. 2007; 93, 651-666. doi: [10.1037/0022-3514.93.4.651](http://psycnet.apa.org/doi/10.1037/0022-3514.93.4.651)

[3] Egger HL, Pine DS, Nelson E, Leibenluft E, Ernst M, Towbin KE, Angold A. NIMH Child Emotional Faces Picture Set (NIMH-ChEFS): A new set of children’s facial emotion stimuli. Int J Methods Psychiatr Res. 2011; 20, 145–156. doi: [10.1002/mpr.343](http://onlinelibrary.wiley.com/doi/10.1002/mpr.343/full)

[4] Wille N, Badia X, Bonsel G, Burström K, Cavrini G, Devlin N, et al. Development of the EQ-5D-Y: a child-friendly version of the EQ-5D. Qual Life Res. 2010; 19: 875–886. doi:[10.1007/s11136-010-9648-y](http://link.springer.com/article/10.1007/s11136-010-9648-y)

[5] Rabin R, de Charro F. EQ-5D: A measure of health status from the EuroQol Group. Ann Med. 2001; 33: 337–343. [doi:10.3109/07853890109002087](http://www.tandfonline.com/doi/abs/10.3109/07853890109002087)

[6] Bouwmans CAM, Schawo S, Hakkaart-van Roijen L. Handleiding Vragenlijst TiC-P voor kinderen. Rotterdam: iMTA, Erasmus Universiteit Rotterdam ([www.imta.nl](http://www.imta.nl)); 2012.

[7] Hakkaart-van Roijen L, Zwirs BWC, Bouwmans C, Tan SS, Schulpen TWJ, Vlasveld L, et al. Societal costs and quality of life of children suffering from attention deficient hyperactivity disorder (ADHD). Eur Child Adolesc Psychiatry. 2007; 16: 316–326. [doi:10.1007/s00787-007-0603-6](http://link.springer.com/article/10.1007/s00787-007-0603-6)

[8] Attkisson CC, Zwick R. The client satisfaction questionnaire: Psychometric properties and correlations with service utilization and psychotherapy outcome. Eval Program Plann. 1982; 5: 233-237. [doi:10.1016/0149-7189(82)90074-X](http://dx.doi.org/10.1016/0149-7189%2882%2990074-X)
