# Supplementary material for: Imagine the bright side of life: A randomized controlled trial of two types of interpretation bias modification procedure targeting adolescent anxiety and depression
Source: PLoS One. 2017 Jul 17;12(7):e0181147. doi: 10.1371/journal.pone.0181147 (PMC5513454; doi:10.1371/journal.pone.0181147)
Supplement: S3 File — S3a, b, and c comprise the complete, original proposal that was reviewed and approved by the ethical committee of the University of Amsterdam. S3d provides the English translation of these documents. (PDF) [file pone.0181147.s003.pdf]

S3 File. Trial protocol. S3a, b, and c comprise the complete, original proposal that was reviewed and approved by the ethical committee of the University of Amsterdam. S3d provides the English translation of these documents.

S3a. Original protocol

S3b. Additional protocol information B1

S3c. Information letters

S3d. Trial protocol translation

UNIVERSITEIT VAN AMSTERDAM

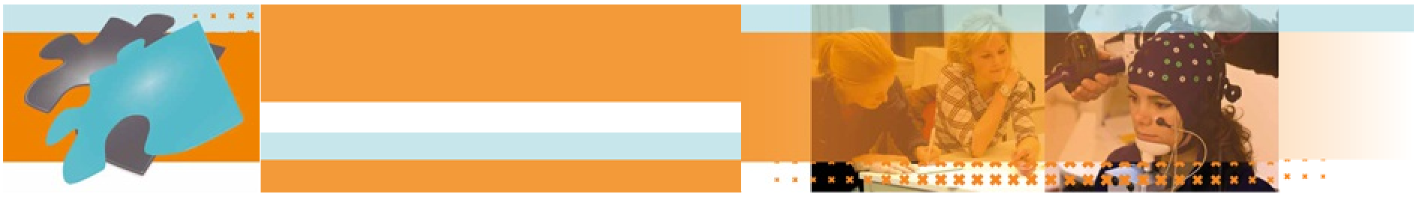

Always look on the bright side of life - Deel II

Project details

**ERB number**	2014-DP-3618
**ID**	3618
**Title**	Always look on the bright side of life - Deel II
**Department**	Developmental Psychology
**Status**	Enter scientific integrity follow up
**Created**	2014-05-12 15:27:38
**Modified**	2014-05-12 15:27:38

Project collaborators

Leone de Voogd	Owner	E.L.deVoogd@uva.nl		

General

Responsible researcher

Leone de Voogd, Elske Salemink, Reinout Wiers

Who conducts the research?

Leone de Voogd & studenten

Research location

Scholen verspreid over Nederland, uitgevoerd vanuit Universiteit van Amsterdam

Brief project description

x

Expected duration of the project

12 maanden

Expected number of participants

3000 stap 1, 300 stap 2

This project is comparable with the following submitted project(number)

2492

Scientific integrity checklist

Who will store and inspect the data?

Leone de Voogd Elske Salemink

When will data collection start (approximately)?

2014-09-01

When will the data collection be completed?

2017-12-31

Is the study exploratory or confirmatory?

exploratory (check this option if the study is both exploratory and confirmatory)

Ethics Checklist

A1. When classifying the research as Medical vs. Non-medical, does it comply with A1, meaning it can be listed under category D (see also Appendix 1, 2.4)?

Yes, it falls into category D

A2. Are consenting adults selected, as described in A2?

Other (or Unsure), explain why in a comment

Het onderzoek vindt plaats bij jongeren tussen 12 en 18 jaar.

Voor stap 1, de screening, worden informatiebrieven verstrekt aan ouders en jongeren en is passieve toestemming vereist.
Voor stap 2, de training, worden informatiebrieven verstrekt aan geselecteerde jongeren en ouders en is van beiden actieve toestemming vereist.

A3. Are participants free to decide to participate and to stop for whatever reason, as listed under A3?

Yes

A4. Are participants subjected to a screening procedure to reduce the risks for adverse effects, as listed under A4?

No,I explain in a comment why

Jongeren worden gescreend op de aanwezigheid van angstige of depressieve klachten als inclusie criterium, omdat bij deze doelgroep het meeste effect verwacht wordt van de training.
Jongeren en hun ouders worden op de hoogte gesteld van de uitslag van de screening.

A5. Is there a risk for chance incidents that should be reported to the participant, as listed under A5?

No, the method precludes chance incidents

A6. Are participants fully informed before participating, and do they sign a consent form, as listed under A6?

Yes, please submit the information letter and the consent form as attachment

A7. Is participant privacy and anonymity guaranteed, as listed under A7?

Yes

A8. In case of deception, does the procedure comply with the conditions listed under A8? (full disclosure concerning risks, accurate debriefing)?

There is no deception

A9. Is there a risk that a substantial number of participants will drop out because the research is considered to be discomforting, as listed under A9?

No

B0. Does the research fully comply with the guidelines for Standard Research?

B1. Gestandaardiseerd onderzoek binnen de afdeling Psychologie

x

Attachments

Add a concise research description (max 1 A4) and any other relevant documents.

1. Toelichting details CE_2014.docx

Enkele details moeten nog gespecificeerd worden. Indien deze in belangrijke mate afwijken van de bijgevoegde beschrijving, zal toestemming voor deze wijziging gevraagd worden.

Scientific integrity follow up

Did you complete the study?

I completed the study, but I am still trying to publish it (option to enter a new end date)

Project history

^ **2015-04-01 11:13:34** Leone de Voogd [by Leone de Voogd]

Zoals op 22/9/2014 aangegeven, loopt het project langer door dan in de oorspronkelijke aanvraag stond.
De voor- en nametingen worden eind april afgerond. De follow-up metingen lopen tot eind november 2015, waarna het volledige project afgerond kan

worden.

2015-04-01 00:01:34 Automated system [by Unknown]

Request Scientific integrity follow up notification send

2014-11-27 16:45:52 Maurits van der Molen [by M.W. van der Molen]

Project approved

2014-11-27 16:45:52 Maurits van der Molen [by M.W. van der Molen]

Dear Colleague,

I hereby acknowledge receipt of the addendum to your project, archived as 2014-DP-3618.
Please use this file number in future correspondence.

The suggested changes have been reviewed. Because it still qualifies as “standard research”, your project is hereby approved.

Any further modifications of the concerning project should be submitted to the Ethics Review Board for evaluation.

Regards,

Maurits van der Molen,
Member of the Ethics Review Board.

2014-11-27 15:44:24 Leone de Voogd [by Leone de Voogd]

(reply to: 18-10-2014 19:47 by Maurits van der Molen)

Graag willen we wederom een wijziging voorleggen in dit project.
In verband met een tegenvallende instroom worden de inclusiecriteria verruimd. I.p.v. de 25% hoogst scorende jongeren zullen de 50% hoogst scorende jongeren worden uitgenodigd. Aangezien het hier ook om jongeren met zeer milde klachten zal gaan, zijn de informatiebrochures overeenkomstig aangepast.
Deze worden per e-mail ingediend (aangezien uploaden nog niet mogelijk is).

2014-10-18 19:47:07 Maurits van der Molen [by M.W. van der Molen]

Project approved

2014-10-18 19:47:07 Maurits van der Molen [by M.W. van der Molen]

Dear Colleague,

I hereby acknowledge receipt of your project, archived as 2014-DP-3618.
Please use this file number in future correspondence.

Your project has been reviewed. Because it qualifies as “standard research”, your project is hereby approved.

Modifications of the concerning project should be submitted to the Ethics Review Board for evaluation.

Regards,

Maurits van der Molen,
Member of the Ethics Review Board.

(reply to: 12-05-2014 19:27 by Maurits van der Molen)

De voorgestelde wijzigingen zijn in goede orde ontvangen, beoordeeld en goedgekeurd.

met vriendelijke groet,

Maurits van der Molen
lid van de ethische commissie Psychologie (UvA0

2014-10-18 19:46:11 Maurits van der Molen [by M.W. van der Molen]

(reply to: 12-05-2014 19:27 by Maurits van der Molen)

De voorgestelde wijzigingen zijn in goede orde ontvangen, beoordeeld en goedgekeurd.

met vriendelijke groet,

Maurits van der Molen
lid van de ethische commissie Psychologie (UvA0

^ **2014-09-22 11:51:06** Leone de Voogd [by Leone de Voogd]

We willen graag de volgende wijzigingen voorleggen t.o.v. de oorspronkelijke CE-aanvraag:

De 25% ipv 20% hoogst scorende jongeren zullen uitgenodigd worden voor training.

Alle trainingen zullen thuis plaats vinden, dus alleen de meting wordt op school gedaan.

Er wordt niet alleen een 3 maanden follow-up gedaan, maar ook nog een na 6 maanden.

Er worden ook oudervragenlijsten afgenomen; over het gedrag van de jongere en over zorggebruik (dit om kosten-effectiviteit te meten).

Data-collectie zal i.v.m. enige uitloop en extra 6 maanden FU meting pas afgerond zijn in september 2015.

Punt A5 – Toevalsbevindingen:
Hoewel jongeren en hun ouders bericht krijgen wanneer zij tot de 25% hoogst scorenden behoren, wordt daarbij alleen aangegeven dat ze 'meer dan gemiddeld' last hebben van emotionele klachten. Daarom zullen wij persoonlijk contact opnemen met jongeren en hun ouders wanneer sprake is van ernstige depressieve of angstklachten (en/of suïcidaliteit) waarbij mogelijk professionele hulp ingeschakeld moet worden.

2014-05-12 19:27:49 Maurits van der Molen [by M.W. van der Molen]

Project approved

2014-05-12 19:27:49 Maurits van der Molen [by M.W. van der Molen]

Dear Colleague,

I hereby acknowledge receipt of your project, archived as 2014-DP-3618.
Please use this file number in future correspondence.

Your project has been reviewed. Because it qualifies as "standard research", your project is hereby approved.

Modifications of the concerning project should be submitted to the Ethics Review Board for evaluation.

Regards,

Maurits van der Molen,
Member of the Ethics Review Board.

2014-05-12 15:27:38 Leone de Voogd [by Leone de Voogd]

Date data ready set to: 01-04-2015

2014-05-12 15:27:38 Leone de Voogd [by Leone de Voogd]

Project submitted for ERB evaluation

2014-04-15 16:55:13 Leone de Voogd [by Leone de Voogd]

Project created

S3b. Additional information B1

(for translation, see S3d)
For information letters and consent form see S3c

Procedure
Fase 1 (passief consent):
Leerlingen uit de geselecteerde klassen krijgen in de klas uitleg over het onderzoek en een informatiefolder. Hun ouders krijgen via school een brief met informatie thuisgestuurd. Wanneer zij bezwaar hebben tegen deelname kan dit bij ons of de school aangegeven worden.
Bij deelname aan fase 1 worden onder schooltijd enkele vragenlijsten ingevuld naar angst- en depressieve klachten. Dit gebeurt groepsgewijs in computerlokalen. Aan het einde van de vragenlijsten wordt gevraagd om adresgegevens wanneer de leerling e.v.t. uitgenodigd wil worden voor fase 2. Leerlingen ontvangen een klein presentje voor deelname (bijvoorbeeld stressbal o.i.d.).

Fase 2 (actief consent):
Leerlingen die in fase 1 bij de 20% hoogst scorende leerlingen behoren (op angst- of depressieve klachten), ontvangen thuis een informatiebrief voor henzelf en hun ouders met een uitnodiging voor fase 2. Bij deze brief zit een toestemmingsformulier, dat door beiden ondertekend teruggestuurd kan worden of op school ingeleverd.
Het onderzoek start met een voormeting inclusief de eerste training op school (60 min, na schooltijd). De overige 7 trainingen (2 per week) worden thuis via het internet gevolgd. Na de 4 weken vindt een nameting plaats op school van wederom 60 minuten. Na 3 maanden wordt nogmaals gevraagd online thuis of op school vragenlijsten in te vullen.
Leerlingen worden beloond d.m.v. cadeaubonnen en deelname aan een loterij. Afhankelijk van hun deelname aan een deel van of alle sessies, kan dit oplopen tot 15 euro aan cadeaubonnen en kans op een prijs zoals een I-pod.

Materialen voor- en nameting fase 2:
Assessmentversies van de aandachtbiastrainingstaak en interpretatietrainingstaak.

Vragenlijsten: angst/depressie, zelfvertrouwen, piekeren, algemene emotionele en gedragsproblemen, aandachtscontrole.

Trainingstaken
Leerlingen worden random toegewezen aan één van zes trainingsgroepen:

1) Aandachtbiastraining
   Deelnemers zoeken een positief gezicht in een 4x4 matrix van negatieve gezichten.
2) Placebo Aandachtbiastraining
   Deelnemers zoeken een 5-bladige bloem in een 4x4 matrix van 7-bladige bloemen.
3) Interpretatietraining
   Deelnemers lezen ambigue scenario's, waarbij zij positieve woordfragmenten aan moeten vullen.
4) Interpretatietraining picture-word versie
   Deelnemers krijgen ambigue plaatjes te zien in combinatie met een positief woord. Zij moeten zich hierbij zo levendig mogelijk een situatie voorstellen.
5) Placebo interpretatietraining.
   Deelnemers lezen neutrale scenario's, waarbij zij neutrale woordfragmenten aan moeten vullen.

6) Test-hertest conditie
   Deelnemers volgen alleen de metingen. Na afloop van de follow-up meting wordt alsnog training 1, 3 of 4 aangeboden.

Deelnemers weten dat van sommige trainingen meer effect wordt verwacht dan andere, maar weten niet welke training zij krijgen.

<u>Stimuli</u>

Bij de verschillende trainingen en metingen van aandachtbias wordt gebruik gemaakt van de volgende emotionele stimuli:

- Blije, bange, boze en verdrietige gezichten uit de NIMH Child Emotional Faces Picture Set.
- Neutrale en boze gezichten uit de NimStim Set en twee gezichten uit de Matsumoto & Ekman set.

Bij de interpretatietraining met plaatjes wordt gebruik gemaakt van foto’s van dagelijkse situaties. Deze plaatjes kunnen zowel positief als negatief geïnterpreteerd worden, maar hebben geen zware emotionele lading. Er zal gedeeltelijk gebruikt gemaakt worden van foto’s die in vergelijkbaar onderzoek in Engeland gebruikt zijn (door de groep van Prof. Emily Holmes).

S3c. Information letters

(in use after change of inclusion criteria (2014-11-27).

[Information letter parent – phase 1 (screening)]

Geachte ouder/voogd,

De school van uw zoon/dochter werkt mee aan een onderzoek van de Universiteit van Amsterdam (UvA) naar de preventie van emotionele klachten bij jongeren: "Always look on the bright side of life".

Graag willen wij u meer informatie geven over de inhoud en achtergrond van dit onderzoek. Voor dit onderzoek is het belangrijk dat zoveel mogelijk jongeren in de leeftijd van 11 t/m 18 jaar deelnemen. De schoolleiding vindt medewerking aan het onderzoek belangrijk en let daarbij ook op de belangen van uw kind. Het onderzoek vindt plaats op school en onder schooltijd.

Uw kind zal binnenkort aan het onderzoek deelnemen, de precieze data vindt u in de brief van school. Indien u niet wilt dat uw kind deelneemt aan dit onderzoek, kunt u dit aangeven bij de onderzoekers (zie onderaan deze brief).

Waar gaat het onderzoek over?

Het onderzoek, uitgevoerd door de afdeling Ontwikkelingspsychologie van de UvA, richt zich op de preventie van emotionele klachten (zoals angstige of depressieve gevoelens) bij jongeren. Ons doel is de kans op het ontstaan van emotionele problemen te verkleinen, beginnende klachten te verminderen en jongeren weerbaarder te maken tegen stress.

Waarom doen we dit onderzoek?

Een groot deel van de middelbare scholieren voelt zich wel eens angstig of somber. Bij zo'n 10-20% van de jongeren zijn deze gevoelens zo sterk dat zij een angststoornis of depressie ontwikkelen. Deze klachten hebben veel gevolgen voor het dagelijks leven van jongeren en voor hun toekomst. Zo gaan emotionele klachten regelmatig samen met slechtere prestaties op school, het gebruik van verslavende middelen of problemen met andere jongeren of de ouders. Het is dan ook erg belangrijk om op tijd hulp te bieden of zelfs te voorkomen dat dit soort klachten ontstaan.

Wat houdt het onderzoek in?

In het eerste deel van het onderzoek, zullen alle leerlingen enkele vragenlijsten invullen over hun gedachten en gevoelens. Dit duurt ongeveer 30 minuten en vindt plaats tijdens de les.

Vervolgens zal een deel van de jongeren uitgenodigd worden voor een onderzoek naar door ons ontwikkelde computertrainingen. Wanneer uw kind bij deze jongeren hoort, sturen wij u opnieuw een informatiebrief. Wij vragen u en uw kind dan zich samen aan te melden voor dit tweede deel van het onderzoek.

Dit onderzoek is goedgekeurd door de Commissie Ethiek van de Afdeling Psychologie van de Universiteit van Amsterdam. Er zijn geen risico's verbonden aan deelname aan dit onderzoek.

Wel of niet meedoen…

Met uw deelname draagt u bij aan het ontwikkelen van een effectief preventieprogramma waar andere jongeren en hun ouders/begeleiders uiteindelijk veel aan kunnen hebben. Mocht uw kind geselecteerd worden voor het vervolgonderzoek, dan bieden wij een training aan

waar hij/zij mogelijk direct baat bij heeft. Daarnaast ontvangen alle kinderen een klein bedankje voor hun deelname aan het onderzoek.

Wat doen we met de gegevens?

Alle gegevens worden strikt vertrouwelijk behandeld en niet aan anderen doorgegeven. De gegevens worden alleen gebruikt om jongeren uit te nodigen voor het tweede deel van het onderzoek en voor eventuele analyse en publicatie in wetenschappelijke tijdschriften. Bij publicatie wordt geen gebruik gemaakt van persoonsgegevens en blijft de anonimiteit van uw kind beschermd. Indien uit de vragenlijsten blijkt dat uw kind zoveel emotionele klachten ervaart dat hier risico's aan verbonden zijn, zullen wij persoonlijk contact opnemen.

Indien u bezwaar heeft…

Deelname aan het onderzoek is geheel vrijwillig. Indien u niet wilt dat uw kind meedoet, kunt u dit laten weten aan de hoofdonderzoeker, Leone de Voogd, via E.L.deVoogd@uva.nl of 020-5256909,In de brief van school staat tot wanneer dit uiterlijk kan.

Als uw kind gedurende het onderzoek niet langer deel wil nemen, kan hij/zij op elk moment stoppen, zonder een reden te hoeven geven. Ook kunt u tot 24 uur na dit onderzoek alsnog uw toestemming intrekken om de gegevens van uw kind te gebruiken. In beide gevallen zullen wij alle gegevens van uw kind uit al onze bestanden verwijderen.

Heeft u nog vragen?

Voor vragen over het onderzoek kunt u contact opnemen met Leone de Voogd, tel: 020-5256909 /
e-mail: E.L.deVoogd@uva.nl. Voor klachten of vragen of opmerkingen die u niet direct met de onderzoeker wilt bespreken, kunt u contact opnemen met dr. Van der Molen lid van de Commissie Ethiek, tel: 020-5256835 / e-mail: M.W.vanderMolen@uva.nl.

Wij hopen dat u en uw zoon/dochter mee willen werken aan dit onderzoek. Hiermee levert u een belangrijke bijdrage aan het voorkomen van emotionele problemen bij jongeren.

Met vriendelijke groet,

Leone de Voogd, MSc. (hoofdonderzoeker)
Dr. Elske Salemink

Programmagroep Ontwikkelingspsychologie
Adres: Weesperplein 4, 1018 XA Amsterdam
Tefeloon: 020-5256909, Email: E.L.deVoogd@uva.nl

NB: Deze brief heeft u via de school van uw zoon/dochter ontvangen. Wij zijn niet in het bezit van persoonlijke gegevens van u of uw kind. Deze ontvangen wij pas van uw zoon/dochter, wanneer deze deelneemt aan dit onderzoek.

[Information letter child – phase 1 (screening)]

Jouw school werkt mee aan een onderzoek van de Universiteit van Amsterdam, "Always look on the bright side of life". Het onderzoek gaat over het verminderen en voorkomen van emotionele problemen bij jongeren, zoals angstige of sombere gevoelens. Jouw klas zal binnenkort aan het eerste deel van dit onderzoek meedoen. In de brief van school lees je wanneer precies. Als je niet mee wilt doen aan dit onderzoek, of als je ouders niet willen dat je hieraan meedoet, dan kunnen jullie dit doorgeven aan de onderzoekers.

Waarom?

Veel jongeren voelen zich wel eens zenuwachtig of een beetje somber. Bijvoorbeeld als ze een spreekbeurt moeten houden, of als ze ruzie hebben met een vriend(in). Sommige jongeren hebben zoveel last van deze gevoelens dat ze het moeilijk vinden om hun schoolwerk te maken of om nog plezier te hebben. Daarom is het belangrijk om op tijd iets te doen aan dit soort gevoelens.

Als je meedoet aan het onderzoek, help je ons een goede training te ontwikkelen, waar andere jongeren later weer iets aan hebben. Mogelijk bieden we jou in de loop van dit onderzoek zo'n training aan. Misschien heb je hier zelf ook iets aan om beter met stress en negatieve gevoelens om te gaan. Als dank voor je deelname krijg je van ons ook een kleinigheidje.

Hoe gaat dat dan?

In het eerste deel van dit onderzoek, zullen we je vragen enkele vragenlijsten in te vullen over je gedachten en gevoelens. Dit duurt ongeveer 30 minuten en vindt plaats tijdens de les.

Vervolgens zullen wij een deel van de jongeren uitnodigen voor deelname aan het tweede deel van het onderzoek. Hierbij onderzoeken we computertrainingen, die mogelijk negatieve gevoelens kunnen verminderen en jongeren kunnen leren om beter met stress of lastige situaties om te gaan. Als jij geselecteerd wordt, zullen we jou en je ouders opnieuw een informatiebrief sturen. Je kunt dan samen met je ouders besluiten of hieraan mee wilt doen en je opgeven.

En als ik nou toch niet mee wil doen….

Je doet vrijwillig mee met dit onderzoek. Als jij of je ouders dit niet willen, dan kunnen jullie dit laten weten aan de hoofdonderzoeker, Leone de Voogd, via E.L.deVoogd@uva.nl of 020-5256909. In de brief van school staat tot wanneer dit uiterlijk kan.
Als je tijdens het onderzoek liever niet meer mee wilt doen, kan dit altijd. Je kunt dit dan tegen de aanwezige onderzoeker zeggen, zonder dat je een reden hoeft te geven.

Hoe zit het met mijn privacy?

De gegevens die jij invult, krijgt niemand anders dan de onderzoekers te zien. Wij gebruiken ze alleen om te kijken wie we uitnodigen voor de trainingen en om te kijken hoe het gaat met hele groepen jongeren. Alleen als blijkt dat je zoveel emotionele klachten hebt dat je risico's loopt, zullen we persoonlijk contact opnemen met jou en je ouders.

Ik heb nog een vraag…

Als je nog vragen hebt, kijk dan eerst met je ouders in de brief die zij gekregen hebben. Als je dan nog meer wilt weten, kunnen jullie contact opnemen met Leone de Voogd, tel: 020-5256909 / email: E.L.deVoogd@uva.nl. Heb je klachten over het onderzoek of vragen die je

niet met de onderzoeker zelf wilt bespreken? Neem dan contact op met dr. Van der Molen lid van de Commissie Ethiek, tel: 020-5256835 / e-mail: M.W.vanderMolen@uva.nl.

We hopen je binnenkort te zien!

Met vriendelijke groet,

Leone de Voogd, MSc. (hoofdonderzoeker)
Dr. Elske Salemink

Programmagroep Ontwikkelingspsychologie
Adres: Weesperplein 4, 1018 XA Amsterdam
Tefeloon: 020-5256909, Email: E.L.deVoogd@uva.nl

[Information letter parent – phase 2 (training)]

Geachte ouder/verzorger,

Uw zoon/dochter heeft kort geleden meegedaan aan het eerste deel van een onderzoek van de Universiteit van Amsterdam (UvA) naar de preventie van emotionele klachten bij jongeren: "Always look on the bright side of life".

Een deel van de jongeren is geselecteerd voor het vervolgonderzoek naar trainingen om negatieve gevoelens te verminderen en te voorkomen. Ook uw kind willen we uitnodigen om mee te doen aan deze trainingen en het bijbehorende onderzoek. Graag willen wij u toestemming vragen voor deelname van uw kind.

Waar gaat het onderzoek ook alweer over?

Het onderzoek, uitgevoerd door de afdeling ontwikkelingspsychologie van de UvA, richt zich op de preventie van emotionele klachten bij jongeren. Ons doel is de kans op het ontstaan van emotionele problemen te verkleinen én jongeren met beginnende klachten te helpen deze te verminderen.

Jongeren die kwetsbaar zijn voor negatieve gevoelens hebben de neiging hun aandacht vooral te richten op negatieve informatie en onduidelijke situaties negatief te interpreteren. Het onderzoek richt zich op het veranderen van deze patronen. Door middel van een computertraining leren jongeren om positiever naar hun omgeving te kijken.

Wat houdt het onderzoek in?

Gedurende vier weken volgen jongeren 2 x per week een training van ongeveer 15 minuten. Zij kunnen deze trainingen thuis via het internet volgen. Hier worden zij aan herinnerd door middel van e-mail en SMS.

Om het effect van de trainingen te meten, vullen de jongeren vragenlijsten in over hun gevoelens en manier van denken en worden diverse computertaken afgenomen. Deze metingen vinden vóór en na de trainingsperiode plaats op school (na schooltijd) en zullen beide keren ongeveer 60 minuten duren.

Drie en zes maanden na afronding van de training zal uw zoon/dochter opnieuw gevraagd worden enkele vragenlijsten in te vullen. Dit vindt in overleg met de school op school of thuis plaats (online).

De planning ziet er dus als volgt uit:

Week 1	Voormeting op school (60 min) en 1e trainingssessie thuis (10-20 min)
Week 2	Twee trainingssessies thuis (2x 10-20 min)
Week 3	Twee trainingssessies thuis (2x 10-20 min)
Week 4	Twee trainingssessies thuis (2x 10-20 min)
Week 5	Eén trainingssessie thuis (10-20 min) en nameting op school (60 min)
Maand 3	Follow-up meting thuis of op school (30 min)
Maand 6	Follow-up meting thuis of op school (30 min)

Uw kind krijgt na aanmelding bericht over de precieze data van de metingen.

Via loting worden leerlingen toegewezen aan een trainingsgroep of een zogenaamde controlegroep. Er is daarbij een kans dat uw kind in eerste instantie geen training of een placebo-training ontvangt, waarvan wij minder of geen effect verwachten. In dat geval zal na afloop van de follow-up meting alsnog de echte training worden aangeboden.

Als uw kind mee mag doen…

Bij deze brief zit een toestemmingsformulier. Zowel u als uw zoon/dochter moeten toestemming geven voor deelname aan het onderzoek. Wanneer u beiden getekend heeft, kunt u het formulier terugsturen in de bijgevoegde retourenvelop.

Als uw kind niet mee mag/wil doen…

Indien u of uw zoon/dochter niet mee wil doen aan het onderzoek, hoeft u het formulier niet terug te sturen. Ook als u of uw kind gedurende het onderzoek niet meer mee wilt doen, kan dit ten alle tijde worden aangegeven bij de aanwezige onderzoekers.

Wat heeft u/uw kind aan het onderzoek?

Het volgen van de trainingen helpt uw kind mogelijk om beter met negatieve gevoelens en stress om te gaan. Daarnaast draagt u bij aan het ontwikkelen van een effectief preventieprogramma waar andere jongeren en hun ouders/begeleiders uiteindelijk veel aan kunnen hebben. Tot slot ontvangen de jongeren cadeaubonnen (tot max. 15 euro voor het gehele onderzoek) voor deelname aan het onderzoek en doen zij mee aan een loterij.

Wat gebeurt er met de gegevens?

Alle gegevens worden strikt vertrouwelijk behandeld en niet aan anderen doorgegeven. De onderzoeksresultaten worden gebruikt om uitspraken te kunnen doen over groepen en er zal dan ook niet gekeken worden naar de individuele resultaten. Bij publicatie wordt geen gebruik gemaakt van persoonsgegevens en blijft de anonimiteit van uw kind beschermd.

Dit onderzoek is goedgekeurd door de Commissie Ethiek van de Afdeling Psychologie van de Universiteit van Amsterdam. Er zijn geen risico's verbonden aan deelname aan dit onderzoek.

Heeft u nog vragen?

Voor vragen over het onderzoek kunt u contact opnemen met Leone de Voogd, tel: 020-5256909 /
e-mail: E.L.deVoogd@uva.nl. Voor klachten of vragen of opmerkingen die u niet direct met de onderzoeker wilt bespreken, kunt u contact opnemen met dr. Van der Molen, lid van de Commissie Ethiek, tel: 0205256835 / e-mail: M.W.vanderMolen@uva.nl.

Wij hopen dat u en uw zoon/dochter mee willen werken aan dit onderzoek. Hiermee levert u een belangrijke bijdrage aan het verminderen en voorkomen van emotionele problemen bij jongeren.

Met vriendelijke groet,

Leone de Voogd, MSc. (hoofdonderzoeker)
Dr. Elske Salemink

Programmagroep Ontwikkelingspsychologie
Adres: Weesperplein 4, 1018 XA Amsterdam
Telefoon: 020-5256909, Email: E.L.deVoogd@uva.nl

[Information letter child – phase 2 (training)]

Beste leerling,

Kort geleden heb je meegedaan aan het eerste deel van een onderzoek van de Universiteit van Amsterdam (UvA) naar het voorkomen en verminderen van emotionele problemen: “Always look on the bright side of life”.

Een deel van de jongeren is geselecteerd voor het vervolgonderzoek. Ook jou willen we graag uitnodigen om onze trainingen te volgen, die gemaakt zijn om negatieve gevoelens te verminderen en te voorkomen. Met deze brief willen we jou vragen of je ook mee wilt doen aan de tweede fase van dit onderzoek.

Waarom ook alweer dit onderzoek?

Veel jongeren voelen zich wel eens zenuwachtig of een beetje somber. Bijvoorbeeld als ze een spreekbeurt moeten houden, of als ze ruzie hebben met een vriend(in). Sommige jongeren hebben zoveel last van deze gevoelens dat ze het moeilijk vinden om hun schoolwerk te maken of om plezier te hebben. Daarom is het belangrijk om op tijd iets te doen aan dit soort gevoelens.

Wat houdt het onderzoek in?

Wij doen onderzoek naar een computertraining, waarin jongeren leren op een positieve manier naar hun omgeving te kijken. Wij verwachten dat dit soort trainingen helpen om beter met stress en negatieve gedachten en gevoelens om te gaan.

Als je meedoet aan dit onderzoek, zal je 4 weken lang 2x per week een computertraining volgen van ongeveer 15 minuten. Dit doe je thuis via het internet op een moment dat het jou uitkomt. We zullen je hieraan herinneren per e-mail en SMS.

Vóór en na de trainingsperiode vul je op school verschillende vragenlijsten in over je gevoelens en gedachten. Ook doe je dan verschillende computertaakjes. Dit duurt beide keren ongeveer 60 minuten. Drie en zes maanden na afloop van de training vragen we je nogmaals op school of thuis via internet een aantal vragenlijsten in te vullen.

De planning ziet er dus als volgt uit:

Week 1	Voormeting op school (60 min) en 1e trainingssessie thuis (10-20 min)
Week 2	Twee trainingssessies thuis (2x 10-20 min)
Week 3	Twee trainingssessies thuis (2x 10-20 min)
Week 4	Twee trainingssessies thuis (2x 10-20 min)
Week 5	Eén trainingssessie thuis (10-20 min) en nameting op school (60 min)
Maand 3	Follow-up meting thuis of op school (30 min)
Maand 6	Follow-up meting thuis of op school (30 min)

Via loting wordt bepaald welke training je ontvangt. Er is daarbij ook een kans dat je eerst helemaal geen training of een ‘nep’-training krijgt. In dat geval kun je na afloop van de follow-up meting alsnog de ‘echte’ training volgen.

En wat heb jij eraan?

Als je meedoet aan het onderzoek, leer je misschien wel beter met stress en negatieve gevoelens om te gaan. Ook help je ons een goede training te ontwikkelen, waar andere jongeren later weer iets aan hebben.

Om je hiervoor te bedanken, krijg je cadeaubonnen voor je deelname aan het onderzoek. Als je álle trainingen en metingen meedoet, kun je 15 euro verdienen. Daarnaast doe je mee aan een loterij waarin je leuke prijzen kunt winnen.

En nu?

Als je mee wilt doen aan het onderzoek, moet je samen met je ouders/verzorgers het toestemmingsformulier invullen en terugsturen. Als je niet mee wilt doen, hoef je helemaal niets te doen. Als je tijdens het onderzoek liever niet meer mee wilt doen, kan dit altijd. Je kunt dit dan tegen de aanwezige onderzoeker zeggen.

Oh en nog één ding…

De gegevens die jij invult, krijgt niemand anders dan de onderzoekers te zien. Wij kijken alleen naar de resultaten van verschillende groepen jongeren.

Ik heb nog een vraag…

Als je nog vragen hebt, kijk dan eerst met je ouders in de brief die zij gekregen hebben. Als je dan nog meer wilt weten, kunnen jullie contact opnemen met Leone de Voogd, tel: 020-5256909 / email: E.L.deVoogd@uva.nl. Heb je klachten over het onderzoek of vragen die je niet met de onderzoeker zelf wilt bespreken? Neem dan contact op met dr. Van der Molen, lid van de Commissie Ethiek, tel: 0205256835 / e-mail: M.W.vanderMolen@uva.nl.

We hopen van je te horen!

Met vriendelijke groet,

Leone de Voogd, MSc. (hoofdonderzoeker)
Dr. Elske Salemink

Programmagroep Ontwikkelingspsychologie
Adres: Weesperplein 4, 1018 XA Amsterdam
Tefeloon: 020-5256909, Email: E.L.deVoogd@uva.nl

Informed consent form phase 2 (training)

Toestemmingsverklaring

Deze verklaring dient door zowel de leerling als de ouder/verzorger ingevuld en ondertekend te worden!

Persoonlijke gegevens leerling

Naam:<br>M / V
School:
Klas:
Geboortedatum:
Telefoon:
E-mail adres:
E-mail adres ouder/verzorger:

Toestemming door leerling

Ja, ik doe mee aan het onderzoek ‘Always look on the bright side of life’ van de Universiteit van Amsterdam. Ik heb de informatie over het onderzoek gelezen en begrepen en weet dat ik mijn toestemming kan intrekken en mijn deelname kan stoppen wanneer ik maar wil.

Datum: Handtekening leerling:

Plaats:

Toestemming door ouder/verzorger

Ja, ik geef mijn zoon/dochter toestemming om mee te doen aan het onderzoek ‘Always look on the bright side of life’ van de Universiteit van Amsterdam. Ik heb de informatie over het onderzoek gelezen en begrepen. Ik heb te allen tijde het recht om mijn toestemming in te trekken en de deelname van mijn zoon/dochter aan het onderzoek te laten stoppen.

Naam ouder/verzorger:

Datum: Handtekening ouder/verzorger:

Plaats:

S3d Trial protocol translation

Ethical Committee Proposal 2014-DP-3618

Project title
Always look on the bright side of life - Deel II

Project collaborators
Leone de Voogd Owner E.L.deVoogd@uva.nl

Responsible researcher
Leone de Voogd, Elske Salemink, Reinout Wiers

Who conducts the research?
Leone de Voogd & students

Research location
Schools in the Netherlands, main research location Amsterdam

Expected duration of the project
12 months

Expected number of participants
3000 phase 1, 300 phase 2.

This project is comparable with the following submitted project(number)
2012-DP-2492

Scientific integrity checklist
Who will store and inspect the data?
Leone de Voogd Elske Salemink

When will data collection start (approximately)?
2014-09-01

When will the data collection be completed?
2017-12-31

Is the study exploratory or confirmatory?
exploratory (check this option if the study is both exploratory and confirmatory)

A1. When classifying the research as Medical vs. Non-medical, does it comply with A1, meaning it can be listed under category D (see also
Appendix 1, 2.4)?
Yes, it falls into category D

A2. Are consenting adults selected, as described in A2?
The study will be conducted with adolescents aged 12 to 18 years. For phase 1, the screening, adolescents and parent receive information letters (see S3c) and passive consent is

required. For phase 2, the training, adolescents and parents receive information letters again (see S3c), and active consent is required from both the adolescent and a parent.

A3. Are participants free to decide to participate and to stop for whatever reason, as listed under A3?
Yes

A4. Are participants subjected to a screening procedure to reduce the risks for adverse effects, as listed under A4?
No. Adolescents are screened on anxiety and depressive symptoms as an inclusion criterion, as the strongest training effects are expected for this population. Adolescents and parent will be debriefed about the results of the screening.

A5. Is there a risk for chance incidents that should be reported to the participant, as listed under A5?
No, the method precludes chance incidents.

A6. Are participants fully informed before participating, and do they sign a consent form, as listed under A6?
Yes, please submit the information letter and the consent form as attachment (see S3c).

A7. Is participant privacy and anonymity guaranteed, as listed under A7?
Yes

A8. In case of deception, does the procedure comply with the conditions listed under A8? (full disclosure concerning risks, accurate debriefing)?
There is no deception

A9. Is there a risk that a substantial number of participants will drop out because the research is considered to be discomforting, as listed under A9?
No

B0. Does the research fully comply with the guidelines for Standard Research?
B1. Standard research within the department of Psychology.

Attachments
B1 Add a concise research description (max 1 A4) and any other relevant documents.
Some details will be further specified or might change. Approval will be requested for substantial changes.

Research description:

<u>Procedure</u>
Phase 1 (passive consent):
Adolescents from selected school classes receive oral information in class and an information letter about the study. Parents receive an information letter at home via school. When the adolescent and/or parent does not want to participate, they can inform either the school or the main researcher.
When participating in phase 1, adolescents will complete questionnaires about anxiety and depressive symptoms. These are administered during school hours in group format in

computer rooms. At the end of the assessment, adolescents are asked to provide their address if they would like to be invited for the training study. Adolescents receive a small present to thank them for their participation.

Phase 2 (active consent):
Adolescents scoring in the upper 20% (of anxiety or depressive symptoms), receive an information letter at home to invite them for the training study. In addition, they receive an informed consent form. Both the adolescent and a parent has to sign and the form can be sent to the University or handed in at school.
The training study will start with an assessment including the first training session at school (60 min, after school hours). The other 7 training sessions (twice a week) will be completed at home via the internet. After 4 weeks, the post-training assessment of 60 minutes is completed at school. After 3 months, follow-up questionnaires will be completed either at home or at school (online).
Adolescents will be rewarded with vouchers and participation in a lottery. Depending on their participation in some or all sessions, this may reach a total amount of 15 euros and the chance to win an I-pod.

<u>Assessment materials:</u>
Assessment versions of the attentional bias training task and interpretation bias training task.

Questionnaires: anxiety/depression, self-esteem, worry, general emotional and behavioral problems, attentional control.

<u>Training tasks</u>
Participants will be randomly allocated to one of six training groups:

1) *Attentional bias training*
   Participants search for one positive face in a 4x4 matrix of negative faces
2) *Placebo Attentional bias training*
   Participants search for a 5-petaled flower in a 4x4 matrix of 7-petaled flowers
3) *Interpretation training*
   Participants read ambiguous scenarios and complete positive word-fragments
4) *Interpretation training picture-word version*
   Participants view ambiguous pictures combined with positive words. They have to imagine this situation as vividly as possible..
5) *Placebo Interpretation training*
   Participants read neutral scenarios and complete neutral word-fragments
6) *Test-retest condition*
   Participants only complete the assessments. After completing follow-up, they are offered training 1, 3, or 4.

Participants know that more effects are expected from some training tasks than from others, but do not know which kind of training they receive.

<u>Stimuli</u>
For the training and assessment of attentional bias, the following stimuli will be used:

- *Happy, fearful, angry and sad faces from the NIMH Child Emotional Faces Picture Set.*

- *Neutral and angry faces form the NimStim Set and two faces from the Masumoto & Ekman set.*

For the interpretation training with pictures, photos of daily situations are used. These pictures can be interpreted both positively and negatively, but are not heavily emotionally laden. Part of the pictures that are used have been used in comparable studies in the UK (by the group of Prof. Emily Holmes).

Proposed changes on 2014-09-22 11:51:06
We would like to propose the following changes:
Instead of 20%, the 25% highest scoring adolescents will be invited for training. All training sessions will be completed at home, and only assessments will be completed at school. In addition to the 3 months follow-up, a 6 months follow-up assessment will be included. Also, parent questionnaires will be administered: about adolescents behavior and use of health care provisions (to assess cost-effectiveness).
Data collection will be completed in September 2015, given the longer follow-up period.
A5 – change findings:
Although adolescents and parents recieve an invitation if the adolescent belongs to the 25% highest scoring participants, they only get an indication that they show 'a more than average level of emotional complaints'. Therefore, we will personally contact those adolescents (and their parents) who display severe levels of anxiety or depression (and/or suicidal thoughts), for whom professional help might be necessary.
Approved on 2014-10-18 19:46:11

Proposed changes on 2014-11-27 15:44:24
We would like to propose another change:
Given low response rates, the inclusion criteria will be changed. Instead of the 25% highest scoring adolescents, the 50% highest scoring adolescents will be invited for training. Since now also adolescents with few complaints will included, the information letters have been changed accordingly.
Approved on 2014-11-27 16:45:52

Scientific integrity follow up
Did you complete the study?
I completed the study, but I am still trying to publish it (option to enter a new end date)
